# Social determinants of health and health inequities in breast cancer screening: a scoping review

**DOI:** 10.3389/fpubh.2024.1354717

**Published:** 2024-02-07

**Authors:** Vama Jhumkhawala, Diana Lobaina, Goodness Okwaraji, Yasmine Zerrouki, Sara Burgoa, Adeife Marciniak, Sebastian Densley, Meera Rao, Daniella Diaz, Michelle Knecht, Lea Sacca

**Affiliations:** ^1^Charles E. Schmidt College of Medicine, Florida Atlantic University, Boca Raton, FL, United States; ^2^Charles E. Schmidt College of Science, Boca Raton, FL, United States

**Keywords:** social determinants of health, breast cancer screening, mammography, health inequities, underserved women, United States

## Abstract

**Introduction:**

This scoping review aims to highlight key social determinants of health associated with breast cancer screening behavior in United States women aged ≥40  years old, identify public and private databases with SDOH data at city, state, and national levels, and share lessons learned from United States based observational studies in addressing SDOH in underserved women influencing breast cancer screening behaviors.

**Methods:**

The Arksey and O’Malley York methodology was used as guidance for this review: (1) identifying research questions; (2) searching for relevant studies; (3) selecting studies relevant to the research questions; (4) charting the data; and (5) collating, summarizing, and reporting results.

**Results:**

The 72 included studies were published between 2013 and 2023. Among the various SDOH identified, those related to socioeconomic status (*n* = 96) exhibited the highest frequency. The Health Care Access and Quality category was reported in the highest number of studies (*n* = 44; 61%), showing its statistical significance in relation to access to mammography. Insurance status was the most reported sub-categorical factor of Health Care Access and Quality.

**Discussion:**

Results may inform future evidence-based interventions aiming to address the underlying factors contributing to low screening rates for breast cancer in the United States.

## Introduction

The social determinants of health (SDOH) are factors outside of the realm of medicine that impact health outcomes and quality of life on a daily basis (1). According to the World Health Organization (WHO), SDOH are defined as “the conditions in which people are born, grow, work, live, and age, and the wider set of forces and systems shaping the conditions of daily life (1).” These determinants of health can be divided into five categories: economic stability, education access and quality, health care access and quality, neighborhood and built environment, and social and community context (2). While factors within each of these categories can individually impact a different facet of a person’s health, these categories often also work collectively to create facilitators and barriers to healthy behaviors and health outcomes (1–3). Such SDOH play a significant role in creating new and worsening existing healthcare disparities and may exhibit a stronger influence on health and well-being than the care received by providers and healthcare organizations (4).

One of the most influential roles of SDOH lies within the realm of equitable access to cancer care (4–7). Specifically, when considering breast cancer, there is significant evidence that supports the influence of SDOH on screening. Despite the presence of innovative screening and treatment strategies, breast cancer remains the second most common type of cancer and is a leading cause of disability and mortality in the United States (8). Breast cancer screening, through mammography and clinical breast examination, is the method of primary prevention that is recommended by the United States Preventive Service Task Force (9). However, research studies showed that health disparities persist, as minority women within the United States are less likely to take advantage of breast cancer screening methods (10–14). Though these studies assessed primarily the role of race and ethnicity on breast cancer screening behaviors, they all found that reported associations were mediated by other SDOH such as quality of health care, education, family income, and health insurance (11–14). Hence, there is a need to explore and understand which determinants act as significant influential factors contributing to low breast cancer screening behaviors. This scoping review aims to highlight key SDOH associated with breast cancer screening behavior in United States women aged ≥40 years old, identify public and private databases with SDOH data at city, state, and national levels, and share lessons learned from United States based observational studies in addressing SDOH in underserved women influencing breast cancer screening behaviors. Findings can guide researchers, physicians, and community workers in improving accessibility, affordability, and quality of breast cancer screening opportunities through culturally competent strategies tailored to satisfy the needs of the at-risk female population group.

## Methods

The review team consisted of a multidisciplinary team of health professionals with extensive knowledge on the role of SDOH in minority populations. The Preferred Reporting Items for Systematic reviews and Meta-Analyses extension for Scoping Reviews (PRISMA-ScR) was utilized as a reference checklist for the sections of this study (15). The Arksey and O’Malley (16) York methodology was used as guidance for this review. This framework employs five steps: (1) identifying research questions; (2) searching for relevant studies; (3) selecting studies relevant to the research questions; (4) charting the data; and (5) collating, summarizing, and reporting results (16). These methods ensure transparency, permits replicability of the search strategy, and increases the reliability of study findings.

### Step 1: identifying research questions

Three research questions were used for this scoping review: (1) What are the major SDOH hindering breast cancer screening in United States women aged > = 40?; (2) What were the major databases/data sources used to capture SDOH data to assess its influence on breast cancer screening behaviors in United States women?; and (3) What are the lessons learned for future recommendations to address SDOH in underserved women at-risk for the disease?

### Step 2: searching for relevant articles

Keywords and MeSH terms were developed in collaboration with a research librarian (MK) who is an expert in scoping review protocols. Search terms included: *breast cancer, breast cancer screening, mammography, race/ethnicity, education level, income, housing instability, insurance coverage, language preferences, health equity, health disparities,* and *medically underserved communities*, among others. Four electronic databases (PubMed, Embase, Web of Science, and Cochrane) were selected due to their breadth and focus on psychosocial and behavioral aspects of chronic illnesses. These databases were searched to identify peer-reviewed literature from primary data sources, secondary data sources, and case reports. The review of the literature was completed over a period of 3 months, from January 2023 to March 2023. The screening of these articles was carried out by senior author (LS) and co-authors (VJ, DL, GO, YZ, SB, AM, SD, MR, and DD).

#### Inclusion criteria

The articles that were included were peer-reviewed observational studies, published in English between 2013 and 2023 that focused on the SDOH, including race/ethnicity, employment, education, food security, insurance status, housing, and access to quality healthcare. These observational studies specifically focused on assessing the significance of the role of SDOH in creating health inequities in breast cancer screenings, particularly for women who are 40 years or older, and are at-risk or have been diagnosed with breast cancer. The ≥40 years old age cut-off was selected based on the American Cancer Society recommended guidelines for screening, which highlight that (1) women between 40 and 44 have the option to start screening with a mammogram every year; (2) women 45–54 should get mammograms every year; and (3) women 55 and older can switch to a mammogram every other year, or they can choose to continue yearly mammograms (17).

#### Exclusion criteria

Excluded studies encompass narrative, scoping, and systematic reviews, as well as qualitative, descriptive, and experimental studies. Additionally, articles were excluded if they did not focus on SDOH as influential factors of breast cancer screening behavior, were assessing knowledge and attitudes rather than exploring SDOH as influencing factors of breast cancer screening, were discussing interventions addressing low breast cancer screening rates and associated disparities that might be related to SDOH, were focusing on survival and mortality rather than screening, and were looking at guideline adherence rather than breast cancer screening behavior itself. Datasets with data collected prior to 2005 were not included in the review.

### Step 3: selecting studies relevant to the research questions

All co-authors (VJ, DL, GO, YZ, SB, AM, SD, MR, and DD) extracted, summarized, and tabulated the data from relevant studies. The senior author (LS) reviewed all tabulated data for accuracy and to resolve any discrepancies. Summary tables included an evidence table (Table 1) describing study characteristics, types of SDOH, and outcomes. Types of SDOH were first listed and then categorized based on Healthy People 2030 into five categories: Economic Stability, Education Access and Quality, Health Care Access and Quality, Neighborhood and Built Environment, and Social and Community Context (18). The Healthy People 2030 is a set of science-based objectives with targets to monitor progress and motivate and focus action (18). The Healthy People 2030 first introduced SDOH objectives in 2010, following the World Health Organization’s (WHO) call to address SDOH to maintain health and quality of life (18). The five categories listed reflect the social conditions and environments that are shaped by a wider set of forces and influence behavioral outcomes (18).

**Table 1:** Study characteristics.

Article #	Primary Author/Year	Study design	Sample size	Study population	Age range	Study purpose	Type of SDOH	SDOH category based on HP 2030	Association between SDOH and Outcome (Significant/non-significant)^*^	Type of methodology/Analysis used
1	Agenor et al. (2020)	Cross-sectional study	*n* = 45,031	National Health Interview Survey Female Respondents	40–75 years old	To examine odds in receiving a mammogram in relationship to sexual orientation across racial/ethnic groups	Race/Ethnicity	Social and community context	Significant	Adjusted Wald tests, Logistic regression
							Sexual orientation	Social and community context	Significant	
2	Agrawal et al. (2021)	Cross-sectional study	*n* = 919	African American church going women from Houston, Texas	40–86 years old	To examine factors associated with adherence to the National Comprehensive Cancer Network breast cancer screening guidelines	Race/Ethnicity	Social and community context	Significant	T-test, Chi-square, Logistic regression
3	Alabdullatif et al. (2022)	Cross-sectional study	*n* = 94,290	National Health Interview Survey female respondents	≥40 years old	To examine the association between IT based health care communication and mammography utilization as modified by race/ethnicity/age	Race/Ethnicity	Social and community context	Significant	Logistic regression, Trend analysis
							Age			
4	Alatrash et al. (2021)	Cross-sectional study	*n* = 316	Muslim and Christian Arab American Women from Jordan, Lebanon, and Egypt	≥40 years old	To examine associations of sociodemographic characteristics with perceived benefits and barriers to mammogram screening	Race/Ethnicity	Social and community context	Significant	Fishers exact test, Bonferroni *post hoc* test, Chi-square test, and OR test
5	Anderson et al. (2014)	Cross-sectional study	*n* = 138	Central cancer registry data linked to Medicare claims from three Appalachian states (Pennsylvania, Ohio, and Kentucky)	≥65 years old	To examine the relationship of an area-based measure of breast cancer screening and geographic area deprivation on the incidence of later stage breast cancer across a diverse region of Appalachia	Economic status	Economic stability	Significant	Exploratory spatial data analysis, multivariate regression, and linear regression
							Insurance status	Health care access and quality	Significant	
6	Asgary et al. (2014)	Cross-sectional study	*n* = 100	Homeless women that received services at Barbara Kleinman Shelter in Brooklyn and Bowery Residence Committee’s Safe Haven at least three times between 2010 and 2012	50–74 years old	To evaluate and compare rates and predictors of mammograms in homeless and low-income domicile patients	Income	Economic stability	Non-significant	T-test, Multivariable logistic regression
							Insurance status	Health care access and quality	Non-significant	
							Housing	Neighborhood and built environment	Non-significant	
							Race	Social and community context	Non-significant	
							Age	Social and community context	Non-significant	
							Access to provider counseling	Health care access and quality	Significant	
							History of mental illness	Social and community context	Non-significant	
							Substance/alcohol abuse	Social and community context	Non-significant	
							HIV status	Social and community context	Non-significant	
7	Ayanian et al. (2013)	Cross-sectional study	*n* = 577,316	Medicaid beneficiaries in 2009	65–69 years old	To examine use of mammography in relation to race/ethnicity in Medicare health maintenance organizations, PPO, and traditional Medicare	Income	Economic stability	Significant	Logistic regression
							Insurance status	Health care access and quality	Significant	
							Race/Ethnicity	Social and community context	Significant	
							Area of residence	Neighborhood and built environment	Significant	
8	Balazy et al. (2019)	Retrospective Cohort study	*n* = 1,057	Single institution women undergoing breast radiotherapy from 2012 to 2017	56–60 years old	To examine whether non-English speaking patients present at a later stage than their respective English-speaking counterparts and whether language is associated with mammographic screening	Language	Social and community context	Significant	Ordinal logistic regression, Trend analysis
							Race/Ethnicity	Social and community context	Significant	
							Age	Social and community context	Significant	
9	Beaber et al. (2016)	Cohort study	*n* = 3,413	Women from Geisel School of Medicine and Brigham and Women’s Hospital primary care networks from 2011 to 2013	≥40 years old	To evaluate factors influencing when women begin screening after turning 40 years of age within a network of primary care practices	Race/Ethnicity	Social and community context	Non-significant	Kaplan–Meier cumulative incidence, Cox proportional hazards regression
							Access to healthcare providers	Health care access and quality	Significant	
							Health insurance	Health care access and quality	Significant	
							Household income	Economic stability	Significant	
							Zip code	Neighborhood and built environment	Significant	
10	Beaber et al. (2019)	Cohort study	*n* = 51,241	10 PROSPR sites with women receiving first mammograms in 2011	50–74 years old	To evaluate multilevel predictors of non-adherence among screened women	Age	Social and community context	Significant	Logistic regression, Multivariable analysis
							Race/Ethnicity	Social and community context	Significant	
							Zip code	Neighborhood and built environment	Non-significant	
							Median income	Economic stability	Non-significant	
11	Calo et al. (2016)	Cross-sectional study	*n* = 1,541	Participants of 2010 Houston Survey and contextual data from United States Census	40–74 years old	To evaluate associations between area level socioeconomic measures and mammography screening among a racially and ethnically diverse sample of women in Texas	Age	Social and community context	Significant	Chi-square test, Two level random intercept regression model, Bivariate analysis, and Multivariable analyses
							Insurance	Health care access and quality	Significant	
							Income	Economic stability	Significant	
							Education	Education access and quality	Significant	
							Race/Ethnicity	Social and community context	Significant	
							Housing	Neighborhood and built environment	Significant	
12	Castaneda et al. (2014)	Cross-sectional study	*n* = 208	Survey through UCSD health system	≥40 years old	To examine factors associated with mammography screening utilization among middle-aged Latinas	Age	Social and community context	Significant	Exploratory factor analysis, Logistic regression
							Income	Economic stability	Significant	
							Education	Education access and quality	Significant	
							Language	Social and community context	Significant	
							Race/Ethnicity	Social and community context	Significant	
13	Cataneo et al. (2022)	Cross-sectional study	*n* = 22,825	LEP and English-speaking female participants who filled the NHIS survey in 2015	40–75 years old	To evaluate the impact of limited language proficiency in screening for breast cancer	Language	Social and community context	Significant	Linear regression, Chi-square test, and Stepwise multivariate regression analysis
							Income	Economic stability	Significant	
							Insurance	Health care access and quality	Significant	
							Access to primary care providers	Health care access and quality	Significant	
							Race/Ethnicity	Social and community context	Significant	
14	Chandak et al. (2019)	Retrospective cross-sectional study	*n* = 7,673	Women diagnosed with breast cancer between 2008 and 2012 as noted in the Nebraska Cancer Registry	40–70 years old	To examine rural–urban differences in access to breast cancer screening in a predominantly rural Midwestern state in the United States	Geographic location	Neighborhood and community context	Significant	Spatial analysis, Hot spot analysis
							Access to mammography facilities	Health care access and quality	Significant	
							Age	Social and community context	Significant	
15	Christensen et al. (2023)	Retrospective cross-sectional study	*n* = 457,476	5% sample of American Indian and White women receiving Medicare fee-for-service in AZ, CA, NY, MX, OK, and WA	40–89 years old	To examine the impact of urbanicity and income on receiving mammography for American Indian women compared with that for White women	Race	Social and community context	Significant	Multivariable logistic regression analysis, Linear regression
							Income	Economic stability	Significant	
							Neighborhood	Neighborhood and built environment	Significant	
16	Clark et al. (2017)	Cohort study	*n* = 48,234	Women who received digital breast tomosynthesis (DBT) from 22 primary care centers in the Dartmouth-Brigham and Women’s Hospital Population-based Research Optimizing Screening through Personalized Regimens research center (PROSPR)	49–65 years old	To examine DBT trends and estimated associations with insurance type	Insurance type	Health care access and quality	Significant	Descriptive statistics, Repeated measures analysis using generalized estimating equations (GEE)
							Zip code	Neighborhood and built environment	Non-significant	
							Race	Social and community context	Non-significant	
							Neighborhood household income	Neighborhood and built environment/Economic stability	Non-significant	
							Age	Social and community context	Non-significant	
17	Clarke et al. (2019)	Cross-sectional study	*n* = 29,951	Women who participated in the 2005, 2008, 2010, 2013, and 2015 National Health Interview Survey	50–74 years old	To present national estimates of mammography screening among women by nativity, birthplace, and percentage of lifetime living in the United States (U.S.)	Birthplace	Neighborhood and built environment	Non-significant	Descriptive Statistics, Two-sided *t* tests
							Citizenship	Social and community context	Non-significant	
							Length of time in the United States	Social and community context	Non-significant	
							Age	Social and community context	Non-significant	
							Race/Ethnicity	Social and community context	Non-significant	
							Educational attainment	Education access and quality	Non-significant	
							Poverty status	Economic stability	Non-significant	
							Health insurance	Health Care Access and Quality	Non-Significant	
18	Davis et al. (2017)	Cross-sectional study	*n* = 758	Patients presenting to radiology department for routine screening mammography from December 2016 to February 2017	> 40 years old	To clarify why late screening might occur in an at-risk population	Race/Ethnicity	Social and community context	Significant	Descriptive statistics, Univariate logistic regression, and Multivariate logistic regression
							Age	Social and community context	Significant	
							Employment status	Economic stability	Significant	
							Income	Economic stability	Significant	
							Insurance status	Health care access and quality	Significant	
							Access to mammography	Health care access and quality	Significant	
							Education level	Education access and quality	Significant	
19	Dong et al. (2022)	Case–control study	*n* = 33,537	Patients diagnosed with invasive breast cancer from the Ohio Cancer Incidence Surveillance System between 2010 and 2017	40–64 years old	To examine whether there were reductions in geospatial disparities in advanced stage breast cancer at diagnosis in Ohio after Medicaid expansion	Area of residence	Neighborhood and built environment	Significant	Space–time scan statistic in SaTScan
							Household income	Economic stability	Significant	
							Medicaid coverage	Health care access and quality	Significant	
							Education level	Education access and quality	Significant	
							Household vehicle availability	Economic stability/Social and community context	Significant	
							Insurance coverage	Health care access and quality	Significant	
20	Duggan et al. (2019)	Cross-sectional study	*n* = 240	Residents of two adjacent rural counties in Lower Yakima Valley in eastern Washington state who self-identify as Latina or Non-Latina white	≥40 years old	To examine county-level difference, stratified by ethnicity, of predictor of breast-screening utilization in rural underserved communities	Race/Ethnicity	Social and community context	Non-significant	Multivariate logistic regression
							Education level	Education access and quality	Significant	
							Income	Economic stability	Non-significant	
							County of residence	Neighborhood and built environment	Significant	
							Access to clinic	Health care access and quality	Significant	
							Age	Social and community context	Significant	
21	Elkin et al. (2014)	Cross-sectional study	*n* = 1,749	Adult women attending mammography facilities certified by the FDA under the Mammography Quality Standards Act (MQSA) in six states in 2011	≥ 40	To survey certified mammography facilities in CA, CT, GA, IA, NM, and NY regarding wait times for next available screening, availability of evening and weekend appointments and digital mammography, and insurance copayment requirements	Access to mammography facilities	Health care access and quality	Significant	Chi-square tests
							Insurance copayments	Health care access and quality	Significant	
22	Fedewa et al. (2016)	Cross-sectional study	*n* = 18,459	Women aged ≥40 years from the 2008 and 2013 National Health Interview Surveys	≥ 40 years old	To examine changes in nationwide mammography prevalence and physician recommendation among younger (≥ 40) and older (≥ 75) women by insurance and SES before and after the 2009 USPSTF BC screening guidelines	Insurance status	Health care access and quality	Significant (for younger women)	Chi-square tests, Logistic regression models
							Income	Economic stability	Significant (for younger women)	
							Age	Social and community context	Significant (for younger women)	
							Race/Ethnicity	Social and community context	Significant (for younger women)	
							Birthplace	Neighborhood and built environment	Significant (for younger women)	
							Education	Education access and quality	Significant (for younger women)	
23	Flores et al. (2018)	Cohort study	*n* = 9,575	Women who underwent screening mammography in 2005 at Harvard Medical School’s main campus and all affiliated community imaging sites	50–64 years old	To evaluate the association between PCP, contact and longitudinal adherence with screening mammography guidelines over a 10-year period across different racial/ethnic groups	Race/Ethnicity	Social and community context	Non-significant	Generalized estimating equations, Logistic regression, Linear regression, and Wald chunk tests
							Age	Social and community context	Non-significant	
							Primary language	Social and community context	Non-significant	
							Insurance status	Health care access and quality	Significant	
							Level of primary care physician interaction	Health care access and quality	Significant	
24	Guo et al. (2019)	Cohort study	*n* = 3,911	African American participants of the Study on Women’s Health Across the Nation (SWAN)	45–63 years old	To analyze economic, social, and psychological factors associated with African American women’s adherence to the recommended breast cancer screening guidelines during their mid-age period	Age	Social and community context	Significant	Multinomial logistic regression
							Quality of life	Social and community context	Significant	
							Employment	Economic stability	Significant	
							Education	Education access and quality	Significant	
							Family income	Economic stability	Significant	
							Access to healthcare provider	Health care access and quality	Significant	
							Transportation access	Neighborhood and built environment	Significant	
25	Henderson et al. (2015)	Cohort study	*n* = 256,470	Black and white female patients enrolled in the Carolina Mammography Registry from 2005 to 2010	≥ 40 years old	To determine if digital screening mammography performs equally well in black and white women	Race	Social and community context	Non-significant	Computed mammography sensitivity, specificity, and positive predictive value (PPV1), random effects logistic regression model, and Chi-square test
							Education level	Education access and quality	Non-significant	
							Rural/urban area of residence	Neighborhood and built environment	Non-significant	
							Age	Social and community context	Non-significant	
26	Henderson et al. (2020)	Cross-sectional study	*n* = 393,430	Women ages ≥40 years receiving screening mammography across three Breast Cancer Surveillance Consortium registries from 2012 to 2017	≥ 40 years old	To evaluate barriers to receiving health care, focusing on caretaker responsibilities, health insurance and cost, and transportation	Age	Social and community context	Significant	Chi-square tests, Multivariate logistic regression, and Wald test
							Race/Ethnicity	Social and community context	Significant	
							Education	Education access and quality	Significant	
							Family/Personal history of breast cancer	Social and community context	Significant	
							Income	Economic stability	Significant	
							Health insurance costs	Health care access and quality	Significant	
							Internet access	Neighborhood and built environment	Significant	
							Local unemployment rate	Economic stability	Significant	
							English language proficiency	Social and community context/education access and quality	Significant	
27	Henry et al. (2014)	Cross-sectional study	*n* = 5,197	Women who received mammography from 2008 to 2010 according to the Utah Behavioral Risk Factor Surveillance System	40–74 years old	To investigate possible pre-disposing and enabling factors associated with nonadherence to screening guidelines among Utah women 40 years and older using survey data from the Utah Behavioral Risk Factor Surveillance System (BRFSS)	Health care access	Health care access and quality	Non-significant	Descriptive statistics, Bivariate analysis, Wald chi-square tests, and Multivariable logistic regression models
							Age	Social and community context	Significant	
							Health insurance	Health care access and quality	Significant	
							Income	Economic stability	Significant	
							Having a regular physician	Health care access and quality	Significant	
							Travel time to nearest facility	Neighborhood and built environment	Non-significant	
28	Hong et al. (2018)	Cross-sectional study	*n* = 196	Korean American women residing in the Chicago metropolitan area	50–74 years old	To identify the relationship between perceived discrimination, trust, and breast cancer screening adherence specifically among Korean American (KA) women	Perceived discrimination	Social and community context	Non-significant	Multiple logistic regressions, Firth logistic regressions
							Trust in health care providers/health care systems	Social and community context	Significant	
							Cultural beliefs	Social and community context	Non-significant	
29	Hubbard et al. (2016)	Cohort study	*n* = 49,775	Medicare-enrolled women who underwent a screening mammogram within a registered Breast Cancer Surveillance Consortium (BCSC) program	66–75 years old	To investigate the sociodemographic factors influencing adherence to screening mammography among older women	Age	Social and community context	Significant	Multivariable logistic regression, Cox proportional hazards regression, and Kaplan–Meier curves
							Income	Economic stability	Significant	
							Education	Education access and quality	Significant	
							Health Literacy	Education access and quality	Significant	
							Access to healthcare	Health care access and quality	Significant	
							Diversity index	Social and community context	Significant	
							Public transportation expenditures	Neighborhood and built environment	Significant	
30	Jena et al. (2017)	Cohort study	*n* = 95,661	Women with individual-subscriber or employer-supplemented MA insurance provided through Kaiser	≥65 years old	To examine the impact of eliminating cost sharing for screening mammography on mammography rates	Age	Social and community context	Significant	Propensity score method, Multivariate logistic regression
							Race/Ethnicity	Social and community context	Non-significant	
							Insurance status	Health care access and quality	Significant	
							Neighborhood socioeconomic status	Social and community context/Economic stability	Non-significant	
31	Jensen et al. (2022)	Cross-sectional study	*n* = 2,065	Low-income, uninsured, or under-insured women in West Texas who were served by the Access to Breast Care for West Texas (ABC4WT) program	40–49 years old	To identify sociodemographic barriers and determinants for breast cancer screenings, as well as screening outcomes, in low-income, uninsured, or under-insured communities in West Texas	Age	Social and community context	Non-significant	Pearson’s Chi-square test, T-tests, and Multivariate logistic regression analysis
							Race/Ethnicity	Social and community context	Non-significant	
							Monthly income	Economic stability	Non-significant	
							County of residence	Social and community context	Non-significant	
32	Jin et al. (2019)	Cross-sectional study	*n* = 303	Korean American women in the Atlanta metropolitan area	50–80 years old	To investigate the factors linked to mammography screening among Korean American women in the state of Georgia, United States	Health literacy	Education access and quality	Significant	Pearson Chi-square, T-tests, Multiple logistic regression
							Health beliefs	Social and community context	Significant	
							Education	Education access and quality	Significant	
							Age	Social and community context	Significant	
							Income	Economic stability	Significant	
							Insurance status	Health care access and quality	Significant	
33	Johnson et al. (2021)	Case–control study	*n* = 3,271	Idaho residents with ductal carcinoma *in situ* or invasive breast cancer	50–64 years old	To assess the time from breast cancer diagnosis to treatment for women enrolled in Idaho’s Women’s Health Check (WHC) Program compared to other female Idaho residents with breast cancer	Socioeconomic status	Economic stability	Non-significant	Chi-square statistics, Stratified Wilcoxon (Van Elteren) tests, Quantile regression
							Age	Social and community context	Non-significant	
							Race/Ethnicity	Social and community context	Non-significant	
							Census trace poverty	Economic Stability	Non-significant	
34	Kadivar et al. (2016)	Cross-sectional study	*n* = 4,249	Hispanic and non-Hispanic United States-born white women who participated in the National Assessment of Adult Literacy	≥40 years old	To investigate the connection between functional health literacy and mammography utilization among Hispanic women, in comparison to non-Hispanic White women in the United States	Health literacy	Education access and quality	Significant	Chi-square test, MML probit regression model
							Income	Economic stability	Significant	
							Age	Social and community context	Significant	
							Medical insurance	Health care access and quality	Significant	
							Race/Ethnicity	Social and community context	Significant	
35	Kempe et el. (2013)	Retrospective cohort study	*n* = 47,946	Medically insured women who had not undergone a mammogram in the past 24 months	52–69 years old	To identify the various factors such as race/ethnicity, socioeconomic characteristics, and health status of women who were not screened for breast cancer in an insured population	Age	Social and community context	Significant	Poisson regression models
							Race/Ethnicity	Social and community context	Significant	
							Language preference	Social and community context	Significant	
							Insurance	Health care access and quality	Significant	
							Primary care encounters	Health care access and quality	Significant	
							Specialty encounters	Health care access and quality	Significant	
36	Khaliq et al. (2015)	Cross-sectional study	*n* = 250	Hospitalized women	50–75 years old	To explore the sociodemographic and clinical factors associated with non-adherence to breast cancer screening among hospitalized women	Race	Social and community context	Non-significant	Logistic regression, Unpaired *t*-test, and Chi square tests
							Education	Education access and quality	Significant	
							Annual household income	Economic stability	Significant	
							Access to primary care physician	Health care access and quality	Significant	
							Age	Social and community context	Non-significant	
37	Kim et al. (2019)	Retrospective cross-sectional study	*n* = 127,298	Females participating in the American Community Survey and Robert Wood Johnson Foundation 500	50–74 years old	To evaluate disparities in city-level screening mammography utilization and to identify factors that may impact urban screening utilization	Zip Code/Geography	Neighborhood and built environment	Significant	Mann–Whitney U test, Tukey–Kramer multiple comparison correction, and Spearman rank correlation
							Health insurance	Healthcare access and quality	Significant	
							Median income level	Economic stability	Significant	
							Poverty	Economic stability	Significant	
							Race	Social and community context	Significant	
38	Kim et al. (2022)	Cross-sectional study	*n* = 497,600	Females across the United States who participated in the Behavioral Risk Factor Surveillance System in 2012, 2014, 2016, and 2018	50–74 years old	To explore the association between diabetes and mammography screening and whether the association varied between racial, ethnic, and geographical groups	Age	Social and community context	Significant	Logistic regression models
							Race	Social and community context	Significant	
							Ethnicity	Social and community context	Significant	
							Employment	Economic stability	Significant	
							Education	Education access and quality	Significant	
							Zip Code/Geography	Neighborhood and built environment	Significant	
							Median income level	Economic stability	Significant	
							Health care coverage	Healthcare access and quality	Significant	
39	Komenaka et al. (2015)	Cross-sectional study	*n* = 1,664	All female patients seen in the Maricopa Medical Center Breast Clinic in Phoenix, Arizona	≥40 years old	To investigate the relationship of health literacy and screening mammography	Age	Social and community context	Significant	Two-sample *t* test, Fisher’s exact test, and Logistic regression analysis
							Race	Social and community context	Significant	
							Ethnicity	Social and community context	Significant	
							Education	Education access and quality	Significant	
							Employment status	Economic stability	Significant	
							Insurance status	Healthcare access and quality	Significant	
							English as primary language	Social and community context	Significant	
40	Kosog et al. (2020)	Retrospective cross-sectional study	*n* = 1,161	Female patients from a single FQHC in a major metropolitan city (Chicago, IL)	50–74 years old	To identify an association between sociodemographic factors and breast cancer screening adherence in FQHC patients including the homeless	Age	Social and community context	Non-significant	Multivariate logistic regression
							Ethnicity	Social and community context	Non-significant	
							Primary insurance policy	Healthcare access and quality	Significant	
							Homelessness status	Economic stability	Significant	
							Language	Social and community context	Non-significant	
							Race	Social and community context	Non-significant	
41	Lapeyrouse et al. (2017)	Cross-sectional study	*n* = 304	Female Latina participants in 2009–2010 ecological household study	>40 years old	To investigate whether differences in ever having a mammogram exist between Latina border residents by health insurance status, to determine whether those Latinas who reported ever having a mammogram vary by healthcare system, and to investigate the ranking of cost, trust, and familiarity as primary reasons for solely seeking health care in the United States or Mexico	Acculturation	Social and community context	Significant	Frequency statistics, Two-proportion *z*-test, Binary logistic regression, T-tests, and Chi squared tests
							Age	Social and community context	Significant	
							Ethnicity	Social and community context	Significant	
							Education	Education access and quality	Non-significant	
							Income	Economic stability	Non-significant	
							Health insurance status	Healthcare access and quality	Significant	
42	Lawson et al. (2021)	Retrospective cohort study	*n* = 7,047	Females diagnosed with breast cancer in Western Washington state	40–74 years old	To determine factors associated with receipt of screening mammography by insured women before breast cancer diagnosis, and subsequent outcomes	Age	Social and community context	Significant	Multivariable logistic regression analysis, Univariable logistic regression models, Kaplan Meier estimator, Log rank test, and Cox proportional hazards model
							Race	Social and community context	Significant	
							Ethnicity	Social and community context	Significant	
							Zip Code/Geography	Neighborhood and built environment	Significant	
							Socioeconomic Disadvantage	Economic stability	Significant	
43	Lee et al. (2016)	Cross-sectional study	*n* = 799,467	Females who had mammograms performed across five BCSC regional facilities from 2011 to 2012	≥40 years old	To compare on-site availability of advanced breast imaging services between imaging facilities serving vulnerable patient populations and those serving non-vulnerable populations	Race	Social and community context	Non-significant	Adjusted log binomial generalized estimating equations
							Ethnicity	Social and community context	Non-significant	
							Household income	Economic stability	Non-significant	
							Rural/Urban residence, zip code	Neighborhood and built environment	Non-significant	
							Education	Education access and quality	Non-significant	
							Access to mammography facilities	Healthcare access and quality	Non-significant	
44	Lee et al. (2017)	Cross-sectional study	*n* = 168	Korean American females in the Midwest	40–79 years old	To investigate breast cancer screening rates and its associated factors in Korean-American immigrant women	Age	Social and community context	Significant	Hierarchical logistic regression analysis
							Race	Social and community context	Significant	
							Ethnicity	Social and community context	Significant	
							Healthcare accessibility	Healthcare access and quality	Significant	
							Income	Economic stability	Significant	
							Education	Education access and quality	Significant	
							Language	Social and community context	Significant	
							Health care literacy	Healthcare access and quality	Significant	
45	Lee et al. (2021)	Cross-sectional study	*n* = 2,313,118	Females attending Breast Cancer Surveillance Consortium affiliated imaging facilities	40–89 years old	To determine women’s access to and use of DBT screening based on race/ethnicity, educational attainment, and income	Access to DBT	Healthcare access and quality	Significant	Descriptive statistics, Log-binomial regression models, and three-step generalized estimated equations
							Race	Social and community context	Significant	
							Ethnicity	Social and community context	Significant	
							Educational attainment	Education access and quality	Significant	
							Income	Economic stability	Significant	
46	Li et al. (2020)	Cross-sectional study	*n* = 12,639 (NHIS)	Civilian noninstitutionalized women living in United States households	40–74 years old	To identify factors and related inconsistencies associated with mammography use in the entirety of the United States population, as well as between black and white subgroups	Age	Social and community context	Significant	RF analysis; Logistic regression
							Family education	Education access and quality	Significant (NHIS)/Non-Significant (BRFSS)	
							Family annual income	Economic stability	Significant	
			*n* = 169,116 (BRFSS)	Women with telephone access in the United States			Number of children at home	Social and community context	Significant	
							Race (Black)	Social and community context	Significant	
			*n* = 181,755 (total)	Women in the United States without a history of breast cancer			Marital status	Social and community context	Mixed	
							Health insurance status	Health care access and quality	Significant	
							Region	Neighborhood and built environment	Significant	
47	Luo et al. (2021)	Cohort	*n* = 33,320	Female Medicare beneficiaries with an initial diagnosis of breast cancer from 2006 through 2014 in the SEER-Medicare database	67–74 years old	To evaluate the contributions of each tumor biology (histologic grade and hormone receptor status) and healthcare (screening mammography use and time delay from mammography to diagnostic biopsy) factor to racial disparity at breast cancer stage-at-diagnosis between African American and white patients	Race	Social and community context	Significant	Probabilistic graph modeling (PGM) using naïve Bayesian network (NBN)-based contribution analysis
48	Molina et al. (2016)	Cross-sectional study	*n* = 536	Federally qualified health center (FQHC)-based group of United States-based Latinas in western Washington State who have not obtained a mammogram in the past 2 years	42–74 years old	To assess the role of four neighborhood characteristics in knowledge-, psychocultural-, and economic-based barriers to mammography use among Latinas	Block group-level socioeconomic deprivation concentration	Neighborhood and built environment/Education access and quality/Economic stability	Non-significant	Multinomial regression models
							Neighborhood socioeconomic-based segregation	Neighborhood and built environment/Economic stability	Significant	
							Neighborhood Latino-based concentration	Neighborhood and built environment/Social and community context	Significant	
							Neighborhood Latino-based segregation	Neighborhood and built environment/Social and community context	Significant	
							Economic	Economic stability/Health care access and quality	Significant	
49	Monsivais et al. (2022)	Cohort study	*n* = 34,588	Female patients of a large health care network in Washington State who had completed a mammogram between January 1 and December 31 in 2017 or 2018 but did not have a mammogram in the following year	≥50 years old	To assess whether racial and socioeconomic inequities in breast cancer screening widened during the COVID-19 pandemic	Age	Social and community context	Significant	Multivariable logistic regression models
							Insurance status	Health care access and quality	Significant	
							Race or ethnicity	Social and community context	Significant	
							Rural or urban residence	Neighborhood and built environment	Significant	
50	Nair et al. (2022)	Cohort study	*n* = 19,292	BSPAN program participants who had at least one mammogram between 2012 and 2019	40–64 years old	To assess prevalence and correlates of baseline adherence, and longitudinal adherence to screening mammograms using data from the longitudinal BSPAN program	Age	Social and community context	Non-significant	Multivariable logistic regression models; multivariable Cox proportional hazards model; chi-square; independent samples t-test; and sensitivity analysis
							Race or ethnicity	Social and community context	Non-significant	
							Marital status	Social and community context	Significant	
							Urbanization	Neighborhood and built environment	Non-significant	
							Proximity to metro	Neighborhood and built environment	Non-significant	
							Rural	Neighborhood and built environment	Non-significant	
							Language preference	Social and community context	Significant	
							Literacy	Education access and quality	Significant	
							Years lived in the United States	Social and community context	Significant	
51	Onega et al. (2018)	Cross-sectional study	*n* = 46,944	Women visiting one of the 15 primary care practices included in the Dartmouth-Hitchock regional network (in NH) and women’s Hospital primary care network (greater Boston)	40–89 years old	To examine the effect of PCP, practice, and health system-level characteristics and processes on the breast cancer screening metrics of overall percent screened and percent screening past age 75	Race or ethnicity	Social and community context	Significant	Generalized linear mixed effects regression models; variance components analysis
							Insurance status	Health care access and quality	Significant	
							Age	Social and community context	Significant	
52	Oviedo et al. (2022)	Cross-sectional study	*n* = 157	Women without a history of breast disease who self-identified as Filipino living in the United States, recruited through the national officers of the Philippine Nurses Association of America	≥40 years old	To determine factors that influence mammogram adherence in Filipino American women using Andersen’s Behavioral Health Model of Services for Vulnerable Populations as the conceptual framework	Breast cancer literacy	Education access and quality	Non-significant	Andersen’s Behavioral Health Model of Services for Vulnerable Populations; logistics regression models; adjusted odds ratios
							Sociocultural deterrents	Social and community context	Non-significant	
							Cultural beliefs	Social and community context	Non-significant	
							Years lived in the United States	Social and community context	Non-significant	
53	Padela et al. (2015)	Cross-sectional study	*n* = 240	Self-identified Muslim, English-speaking women recruited from 11 CIOGC-affiliated mosques and Muslim organization sites in Greater Chicago	>40 years old	To assess relationships between several religion-related factors and breast cancer screening in a group of Chicago-based Muslim women	Religiosity	Social and community context	Significant	Bivariate testing (ex. unadjusted odds ratios) and multivariate logistic regression models
							Perceived religious discrimination in healthcare	Social and community context	Significant	
							Age	Social and community context	Significant	
							Years of residence in the United States	Social and community context	Significant	
							Ethnicity	Social and community context	Non-significant	
54	Paranjpe et al. (2022)	Retrospective cross-sectional study	*n* = 7,990	Civilian, noninstitutionalized Asian and non-Hispanic white women who completed the National Health Interview Survey	≥40 years old	To determine whether breast cancer screening practices were different between Asian and non-Hispanic white women in a national population-based study	Race	Social and community context	Significant	Taylor series linearization methods; Wald chi-square tests; and Multivariable logistic regression
							Insurance status	Healthcare access and quality	Significant	
							Education	Education access and quality	Significant	
							Family income	Economic stability	Significant	
							Place of Birth in United States	Neighborhood and built environment	Significant	
55	Patel et al. (2014)	Cross-sectional study	*n* = 334	Low-income African American women in Nashville, Chattanooga, and Memphis	≥ 40 years old	To examine socio-demographic factors that influence decision to use mammography and other breast cancer screenings in low-income African Americans and examine differences in obstacles to screening by geographic region	Age	Social and community context	Non-significant	Chi-square test, Binary logistic regression model
							City of residence	Neighborhood and built environment	Significant	
							BMI	Healthcare access and quality	Significant	
							Annual household income	Economic stability	Significant	
							Health insurance status	Healthcare access and quality	Non-significant	
							Transportation access	Neighborhood and built environment	Significant	
							Medical visits in the Past 12 months	Neighborhood and built environment	Non-significant	
							Education	Education access and quality	Non-significant	
							Employment status	Economic stability	Non-significant	
56	Ryu et al. (2013)	Cross-sectional study	*n* = 1,596	Immigrant women in five Asian-American ethnic groups participating in the 2009 California Health Interview Survey	40–70 years old	To compare rates of screening mammography among immigrant women in five Asian-American ethnic groups in California, and ascertain the extent to which differences in mammography rates among these groups are attributable to differences in known correlates of cancer screening	Age	Social and community context	Non-significant	Wald chi-square design-adjusted test of independence, Multiple logistic regression, Predicted probabilities
							English proficiency	Social and community context	Non-significant	
							Educational attainment	Education access and quality	Significant	
							Ethnicity	Social and community context	Significant	
							Income	Economic stability	Non-significant	
							Current health insurance	Healthcare access and quality	Significant	
57	Sabatino et al. (2016)	Cross-sectional study	*n* = 1,429 (2010)	Female Medicare beneficiaries without breast cancer history between 2010 and 2013	65–74 years old	To examine whether mammography use increased after elimination of Medicare cost sharing for screening mammography and whether changes varied for different groups of women	Age	Social and community context	Significant	Pearson Wald F test, Multivariable logistic regression
							Race	Social and community context	Non-significant	
							Ethnicity	Social and community context	Significant	
							Birthplace	Neighborhood and built environment	Non-significant	
			*n* = 2,152 (2013)				Income	Economic stability	Non-significant	
							Access to Care	Healthcare access and quality	Significant	
							Type of health insurance	Healthcare access and quality	Significant	
							Number of provider visits	Healthcare access and quality	Significant	
58	Schommer et al. (2023)	Retrospective cross-sectional study	*n* = 781	Breast cancer female patients from Seton Medical Center Austin tumor registry between March 1, 2019 and March 2, 2021	40–70 years old	To explore the relationship between COVID-19 (before and after) and stage distribution, time-to-intervention, and insurance status of patients presenting with breast cancer in the Austin local cancer center	Age	Social and community context	Significant	Descriptive statistics, Chi-square test, Fisher exact test, unpaired T-test, Wilcoxon signed-rank test, Multinomial Logistic regression, Two-tailed Wald test
							Sex	Social and community context	Non-significant	
							Race	Social and community context	Significant (Pre and Post COVID)	
							Ethnicity	Social and community context	Significant (Pre and Post COVID)	
							Insurance status	Healthcare access and quality	Significant	
							Time from breast cancer diagnosis to first treatment	Healthcare access and quality	Significant	
59	Sealy-Jefferson et al. (2019)	Cross-sectional study	*n* = 7,120	Racially/ethnically diverse post-menopausal women from the Women’s Health Initiative Survey (1993–2014)	50–79 years old	To examine whether rural–urban residence was associated with stage at breast cancer diagnosis among large well-defined racially/ethnically diverse cohort of postmenopausal women	Age	Social and community context	Significant	Univariable logistic regression, Multivariable logistic regression
							Race	Social and community context	Non-significant	
							Ethnicity	Social and community context	Non-significant	
							Education	Education access and quality	Non-significant	
							Rural/Urban Residence, Zip Code	Neighborhood and built environment	Non-significant	
							Social Strain	Social and community context	Non-significant	
							Health insurance status	Health care access and quality	Non-significant	
							Social Support	Social and community context	Non-significant	
60	Selove et al. (2016)	Retrospective cohort Study	*n* = 4,476	Non-Hispanic Black and White non-HMO Medicare women, who resided in United States, who had a mammogram, biopsy, and breast cancer diagnosis during 2005–2008	65–84 years old	Examine the length of critical intervals between abnormal mammogram and breast cancer treatment within a large cohort of Medicare beneficiaries varying by age, race, and medical comorbidities	Age	Social and community context	Significant	Cox proportional hazard models, Logistic regression models
							Race	Social and community context	Non-significant	
							Ethnicity	Social and community context	Non-significant	
							Physical comorbidities	Healthcare access and quality	Significant	
61	Shon et al. (2019)	Cross-sectional study	*n* = 3,710	Immigrant Asian women who filled the 2005,2007, 2009, and 2011 California Health Interview Survey	≥40 years old	To examine significant predictors of never having a mammogram among Chinese, Vietnamese, and Korean immigrant women living in California and age 40 years and older and to explore whether relationships between enabling components and acculturation components and odds of never having a mammogram vary across Chinese, Vietnamese, and Korean immigrant women	Ethnicity	Social and community context	Non-significant	Bivariate analysis (Chi-square or ANOVA), Multivariate logistic regression
							Age	Social and community context	Significant	
							Education	Education access and quality	Non-significant	
							Federal poverty level	Economic stability	Non-significant	
							Age	Social and community context	Non-significant	
							Employment	Economic stability	Non-significant	
							English proficiency	Social and community context	Non-significant	
							Years lived in the United States	Neighborhood and built environment	Non-significant	
							Insurance type	Healthcare access and quality	Non-significant	
							Number of Physician Visits in the past 12 months	Healthcare access and quality	Significant	
							Number of Chronic Illnesses	Healthcare Access and Quality	Non-significant	
62	Spada et al. (2021)	Retrospective cross-sectional study	*n* = 35,735	Female breast cancer patients registered in the Pennsylvania Cancer Registry	50–64 and 68–74	To determine if increased access to health insurance following the Affordable Care Act (ACA) resulted in an increased proportion of early-stage breast cancer diagnosis among women in Pennsylvania, particularly minorities, rural residents, and those of lower socioeconomic status	Health Insurance Access	Healthcare access and quality	Non-significant	T-tests; Multivariable logistic regression models; Difference-in-differences analysis
							Area Deprivation Index	Neighborhood and built environment	Non-significant	
							Race	Social and community context	Significant (for 68–74)	
							Ethnicity	Social and community context	Significant (for 68–74)	
							Area of Residence	Neighborhood and built environment	Non-significant	
							PCP Density	Healthcare access and quality	Non-significant	
63	Tangka et al. (2017)	Cross-sectional study	*n* = 3,821,084	Medicaid-insured women in the United States from 2006 to 2008	40–64 years old	To assess racial/ethnic and geographic disparities in the use of breast cancer screening	Race	Social and community context	Significant	Regression models; Generalized Estimating Equations (GEE)
							Ethnicity	Social and community context	Significant	
							State of residence	Neighborhood and built environment	Significant	
64	Thomas et al. (2018)	Retrospective cohort study	*n* = 14,651	Medicaid-insured women (not dual enrolled) in California who received treatment in the specialty mental health care system and have filled least one antipsychotic prescription	48–67 years old	To examine mammogram disparities for those with severe mental illness and the contribution of psychosocial factors to mammogram use among women with severe mental illness	Healthcare access and utilization	Healthcare access and quality	Significant	Poisson models with robust standard errors
							Health insurance status	Healthcare access and quality	Significant	
							Race	Social and community context	Significant	
							Ethnicity	Social and community context	Significant	
							County of residence	Neighborhood and built environment	Non-significant	
							Age	Social and community context	Non-significant	
65	Tran et al. (2019)	Cross-sectional study	*n* = 482,360	U.S. female survey participants in the 2012, 2014, or 2016 Breast and Cervical Cancer-Screening module of the Behavioral Risk Factor Surveillance System (BRFSS) survey	≥ 40 years old	To explore urban–rural disparities in United States breast cancer screening practices at the national, regional, and state levels	Area of residence (urban/suburban/rural)	Neighborhood and built environment	Significant	Binary logistic regression models
							Age	Social and community context	Significant	
							Race	Social and community context	Significant	
							Education	Education access and quality	Significant	
							Healthcare coverage	Healthcare access and quality	Significant	
							Healthcare access and utilization	Healthcare access and quality	Significant	
66	Vang et al. (2020)	Cross-sectional study	*n* = 518	Medically underserved women in NYC	≥40 years old	To examine the relationship between language preference and screening mammogram adherence	Ethnicity	Social and community context	Significant	Descriptive statistics (Chi-square tests and Fisher’s exact tests), Bivariate analyses and multiple logistic regressions
							Age	Social and community context	Significant	
							Race	Social and community context	Significant	
							Education	Education access and quality	Significant	
							Lack of sufficient healthcare coverage	Healthcare access and quality	Significant	
							Language	Social and community context	Significant	
67	Virk-Baker et al. (2013)	Cross-sectional study	*n* = 406,602	White and Black women in fee-for-service Medicare plans from 203 United States counties with highest risk of breast cancer deaths	65–74 years old	To assess the uptake of breast cancer screening in women 65–74 years old from counties with most of the breast cancer deaths in Black older women	Race	Social and community context	Non-significant	Logistic regression
							Comorbid conditions	Healthcare access and quality	Non-significant	
							Age	Social and community context	Non-significant	
							Education	Education access and quality	Non-significant	
							ER utilization	Healthcare access and quality	Non-significant	
							Economic status	Economic stability	Non-significant	
68	Wang et al. (2018)	Cross-sectional study	*n* = 8,347	Patients cared by Accountable Care Organizations (ACO) clinics in rural Nebraska with average risk of breast cancer	50–74 years old	To understand the adherence to the biennial breast cancer screening guideline by rural women with average risk for breast cancer	Age	Social and community context	Significant	Descriptive statistics, Multiple logistic regression, Spearman correlations, and Generalized estimating equation method
							Gender	Social and community context	Significant	
							Race	Social and community context	Significant	
							Ethnicity	Social and community context	Significant	
							Insurance status	Healthcare access and quality	Significant	
							Preferred language	Social and community context	Significant	
							Travel time to clinic	Healthcare access and quality	Significant	
							County poverty rate	Economic stability	Significant	
							County uninsured rate	Healthcare access and quality	Significant	
							Race/Ethnicity composition of county	Social and community context	Significant	
69	Wiese et al. (2023)	Retrospective study	*n* = 73,718	Female population in the United States with limited accessibility to mammography (living more than 20-min drive time to nearest mammography facility)	45–84 years old	To evaluate the travel-time based geographic accessibility to mammography facilities at the census tract level by urban–rural status in continuous US from 2006 to 2022	Rural vs. Urban/Suburban Setting	Neighborhood and built environment	Non-significant	Descriptive statistics, Regression analysis
							Accessibility to screening facility	Healthcare access and quality	Non-significant	
70	Wilcox et al. (2016)	Cross-sectional study	*n* = 697	Randomly sampled households with at least one female tenant selected through 20 United States census tracts with Haitian population	≥40 years old	To identify the correlation between race/ethnicity and annual mammogram compliance	Age	Social and community context	Significant	Binary logistic regression; Chi-square tests
							Race	Social and community context	Significant	
							Ethnicity	Social and community context	Significant	
							Education level	Education access and quality	Significant	
							Preferred language	Social and community context	Significant	
							Poverty status	Economic stability	Significant	
							Employment status	Economic stability	Significant	
							Insurance coverage	Healthcare access and quality	Significant	
							Provider visits	Healthcare access and quality	Significant	
71	Wilkerson et al. (2023)	Retrospective cohort study	*n* = 738	Female patients who underwent treatment for BC at a quaternary care academic medical center or affiliate zonal hospital	40–45 years old	To discover if the majority of Black women are diagnosed with breast cancer on their first mammogram and to determine if the connection between patient demographics and primary findings of breast cancer are of importance for preventative care	Age	Social and community context	Significant	Chi-square test; multivariate logistic regression; Wilcoxon rank-sum test
							Race	Social and community context	Significant	
							BMI	Healthcare access and quality	Significant	
							Insurance coverage	Healthcare access and quality	Significant	
72	Wu et al. (2021)	Retrospective cohort study	*n* = 1,044	Visually impaired women enrolled in fee-for service Medicare	65–72 years old	To assess whether receiving breast cancer screenings are similar for women w/wo visual impairment	Age	Social and community context	Significant	Chi-square test; Multivariable conditional logistic regression
							Race	Social and community context	Significant	
							Environment	Neighborhood and built environment	Significant	
							Insurance coverage	Healthcare access and quality	Significant	
							Urbanization	Neighborhood and built environment	Significant	

^*^Statistical significance was assessed based on the *p* value (*p* < 0.05).

Significance of associations between breast cancer screening as an outcome and identified SDOH were reported (Table 1). Table 2 included a list of databases from where the data was accessed, the availability status of the data (public/private), and the geographical level from where the data was extracted. Basic qualitative content analysis was carried out to identify similar themes in future directions across studies highlighted in Table 3. The three phases of qualitative content analysis for the results of primary qualitative research described by Elo and Kyngas (19) were applied: (i) preparation, (ii) organizing, and (iii) reporting.

**Table 2:** Database availability status and characteristics.

Primary Author/Year	Database/Data source	Publicly available (yes/no)	City/State/National level
Agenor et al. (2020)	National Health Interview Survey (2013–2017)	Yes	National
Agrawal et al. (2021)	Surveys conducted at Three Texas Churches	No	State
Alabdullatif et al. (2022)	National Health Interview Survey (2011–2018)	Yes	National
Alatrash et al. (2021)	Surveys conducted primarily in Arab American mosques and churches	No	City
Anderson et al. (2014)	National Program of Cancer Registries	Yes	State
Asgary et al. (2014)	EHRs from shelter-based clinics of Lutheran Family Health Centers	No	City
Ayanian et al. (2013)	Medicare beneficiary summary file	Yes	National
Balazy et al. (2019)	EHRs from Stanford Health	No	City
Beaber et al. (2016)	EHRs from Dartmouth-Hitchcock Health System and Brigham and Women’s Hospital	No	City
Beaber et al. (2019)	EHRs from 10 PROPSR research medical facilities	No	National
Calo et al. (2016)	United States Census Bureau and Health of Houston Survey	Yes	City
Castaneda et al. (2014)	Survey from UCSD patients 2007–2008	No	City
Cataneo et al. (2019)	National Health Interview Survey (2015)	Yes	National
Chandak et al. (2019)	Nebraska Cancer Registry (2008–2012)	Yes	State
Christensen et al. (2023)	Medicare Beneficiary Summary File	No	State
Clark et al. (2017)	2013 US Census American Community Survey	Yes	State
Clark et al. (2019)	National Health Interview Survey (2005, 2008, 2010, 2013, 2015)	Yes	National
Davis et al. (2017)	Surveys conducted at the radiology department of the University of Arizona College of Medicine	No	State
Dong et al. (2022)	Ohio Cancer Incidence Surveillance System (OCISS)	No	State
Duggan et al. (2019)	Surveys conducted at grocery stores, religious organizations, and community events	Yes	County
Elkin et al. (2014)	FDA’s searchable online database of facilities	Yes	State
Fedewa et al. (2016)	National Health Interview Survey (2013)	No	National
Flores et al. (2018)	Institution’s Research Patient Data Registry, MagView, Burtonsville, Maryland	No	City
Guo et al. (2019)	Study of Women’s Health Across the Nation (SWAN)	No	National
Henderson et al. (2015)	Carolina Mammography Registry (CMR)	No	State
Henderson et al. (2020)	Breast Cancer Surveillance Consortium (BCSC), a National Cancer Institute (NCI)-funded network of mammography registries across the United States.	No	National
Henry et al. (2014)	The 2008 and 2010 Utah Behavioral Risk Factor Surveillance System	No	State
Hong et al. (2017)	Questionnaires	No	City Level
Hubbard et al. (2016)	Breast Cancer Surveillance Consortium (BCSC)	Yes	National Level
Jena et al. (2017)	Kaiser Permanente MA plans	No	State level
Jensen et al. (2022)	Access to Breast Care for West Texas (ABC4WT)	No	State level
Jin et al. (2019)	Self-report survey questionnaires	No	State level
Johnson et al. (2021)	Cancer Data Registry of Idaho (CDRI)	Yes	State level
Kadivar et al. (2016)	National Assessment of Adult Literacy (NAAL)	Yes	National Level
Kempe et el. (2013)	Kaiser Permanente Colorado (KPCO)	No	State level
Khaliq et al. (2015)	Bedside interviews	No	City
Kim et al. (2019)	American Community Survey and Robert Wood Johnson Foundation 500 Cities Project with data from Behavioral Risk Factor Surveillance System	Yes	City
Kim et al. (2022)	Cross sectional data from 2012, 2014, 2016, and 2018 Behavioral Risk Factor Surveillance System	Yes	National
Komenaka et al. (2015)	Maricopa Medical Center Breast Clinic data	No	City
Kosog et al. (2019)	FQHC Electronic Medical Record	No	City
Lapeyrouse et al. (2017)	2009–2010 Ecological Household Study on Latino Border Residents in El Paso County, TX	No	City
Lawson et al. (2021)	Insurance enrollment data from regional commercial insurers and Medicare liked with records from the Cancer Surveillance System from 2007–2018	No	State and National
Lee et al. (2016)	Breast Cancer Surveillance Consortium	Yes	National
Lee et al., 2017	Baseline data from mobile phone program “mMammogram”	No	State/Regional
Lee et al. (2021)	Breast Cancer Surveillance Consortium	Yes	National
Li et al. (2020)	2016 National Health Interview Survey	Yes	National
	2016 Behavioral Risk Factor Surveillance System	Yes	National
Luo et al. (2021)	SEER Medicare	Yes	National
Molina et al. (2016)	2011–2014 Fortaleza Latina!	Yes	State
Monsivais et al. (2022)	Patient data from MultiCare health system, a large state-wide, non-profit healthcare system with 230 clinics and hospitals across Washington State	No	State
Nair et al. (2022)	2012–2019 electronic health record data for BSPAN program participants	Yes	State
Onega et al. (2018)	PROSPR research centers including the primary care populations of the Dartmouth-Hitchock regional network (in NH) and the Brigham and Women’s Hospital primary care network (in greater Boston)	Yes	National
Oviedo et al. (2022)	Self-administered, web-based surveys sent through the PI’s network of friends and through the national officers of the Philippine Nurses Association of America and further through snowball recruitment	No	National
Padela et al. (2015)	Self-administered surveys given to participants at sites affiliated with the Council of Islamic Organizations of Greater Chicago (CIOGC) in the Chicago metro area	No	City
Paranjpe et al. (2022)	2015 National Health Interview Survey	Yes	National
Patel et al. (2014)	Meharry CNP Community Survey Database	No	State
Ryu et al. (2013)^*^	2009 California Health Interview Survey	Yes	State
Sabatino et al. (2016)	National Health Interview Survey Data	Yes	National
Schommer et al. (2023)	Seton Medical Center Austin Tumor Registry	No	City
Sealy-Jefferson et al. (2019)	Women’s Health Initiative Program (WHI)	No	National
Selove et al. (2016)	Center for Medicare and Medicaid Services (CMS)	No	National
Shon et al. (2019)	California Health Interview Survey data	Yes	State
Spada et al. (2021)	Pennsylvania Cancer Registry	Yes	State
Tangka et al. (2017)	Fee-for-service claims and encounter data from Centers for Medicare and Medicaid Services	No	National
Thomas et al. (2018)	California Medicaid (Medi-Cal) Administrative, Pharmacy, and Billing Systems	No	State
	Client and Service Information System		
Tran et al. (2019)	Behavioral Risk Factor Surveillance System surveys (BRFSS)	Yes	National
Vang et al. (2020)	Participants of breast health education programs at various communities and faith-based organizations in MU areas of NYC	No	City
Virk-Baker et al. (2013)	Medicare claims data for outpatient procedures, physician visits and inpatient stays from 2001–2006	Yes	National
Wang et al. (2018)	Clinic EMRs and provider surveys from an ACO organization	No	State
	Secondary data obtained from Area Health Resource File administered by Health Resources and Services Administration		
Wiese et al. (2023)	US FDA, BRFSS	Yes	National
Wilcox et al. (2016)	US Department of Health	Yes	State
Wilkerson et al. (2023)	U.S Department of Health	Yes	National
	CDC		
	Prevention and National Cancer Institute		
	JNCI		
Wu et al. (2021)	Medicare database	No	National
	Clinical Modification (ICD-9-CM) billing codes		
	Current Procedural Terminology (CPT)		
	Healthcare Common Procedure Coding System (HCPCS)		
Young et al. (2020)	FDA’s mammography facility database	Yes	State
	American Community Survey US Census Rural -Urban community (RUCA) codes		

^*^For one control variable, county-level PCP data were obtained across the state from a different database: Area Health Resources Files.

**Table 3:** Lessons learned identified from thematic analysis across included studies.

Lessons learned themes
Lack of health insurance was strongly associated with lower breast cancer screening rates across various populations. Functional health literacy was found to be significantly associated with mammography receipt; however, the relationship between health literacy and mammography can be influenced by factors such as ethnicity and language-preference acculturation. Economic factors such as poverty level was a strong indicator of breast cancer screening rates. Geographic factors including regional poverty are associated with increased late-stage breast cancer and lower breast cancer screening rates. Rural areas were associated with less access to on-site breast cancer screening access and had lower overall breast cancer screening rates. Women who identified themselves as nonwhite ethnicity, with the exception of Asians, had a higher likelihood of being unscreened. Asian women with less time spent in the U.S. and Korean populations had lower screening rates due to limited acculturation, lack of education surrounding breast cancer screening, and lack of insurance. There is a need to address culturally specific barriers, such as distrust of physicians, which may increase Black women’s confidence in breast cancer screenings and motivation to have preventive breast cancer care. Methods to enhance patient–provider communication may be important to increasing adherence to mammogram screening guidelines for those reporting less than ideal interactions with healthcare providers. The COVID-19 pandemic was correlated with lower screening rates in women, possibly due to limited healthcare access for individuals. Breast cancer screening and adherence rates differed depending on the religious values of certain populations, more specifically, fatalism-emphasizing religions led to less screening adherence. Cultural efforts include developing culturally appropriate interventions and training health professionals in culturally competent communication skills, while structural efforts include removing barriers to access, improving health insurance coverage, language proficiency, and transportation services. Community-tailored educational campaigns to reinforce the importance of establishing yearly mammogram screening behaviors can be powerful and effective tools for increasing adherence across various populations. Facilitating access to IT may help increase mammography utilization, which may contribute to eliminating disparities in breast cancer mortality.

### Step 4 and 5: charting the data and collation, summarization, and reporting of results

Study characteristics were tabulated for primary author/year, study design, sample size, study population, age range, study purpose, type of SDOH, SDOH category based on HP 2030, association between SDOH and outcome (significant/non-significant), and type of methodology/analysis used for data analysis (Table 1). Identified databases were tabulated by primary author/year, database/data source, public availability, and city/state/national level (Table 2). Each database was stratified based on availability (publicly available/not publicly available) and location (city/state/national level). Lessons learned from each relevant study were highlighted in Table 3.

## Results

**Figure 1:**
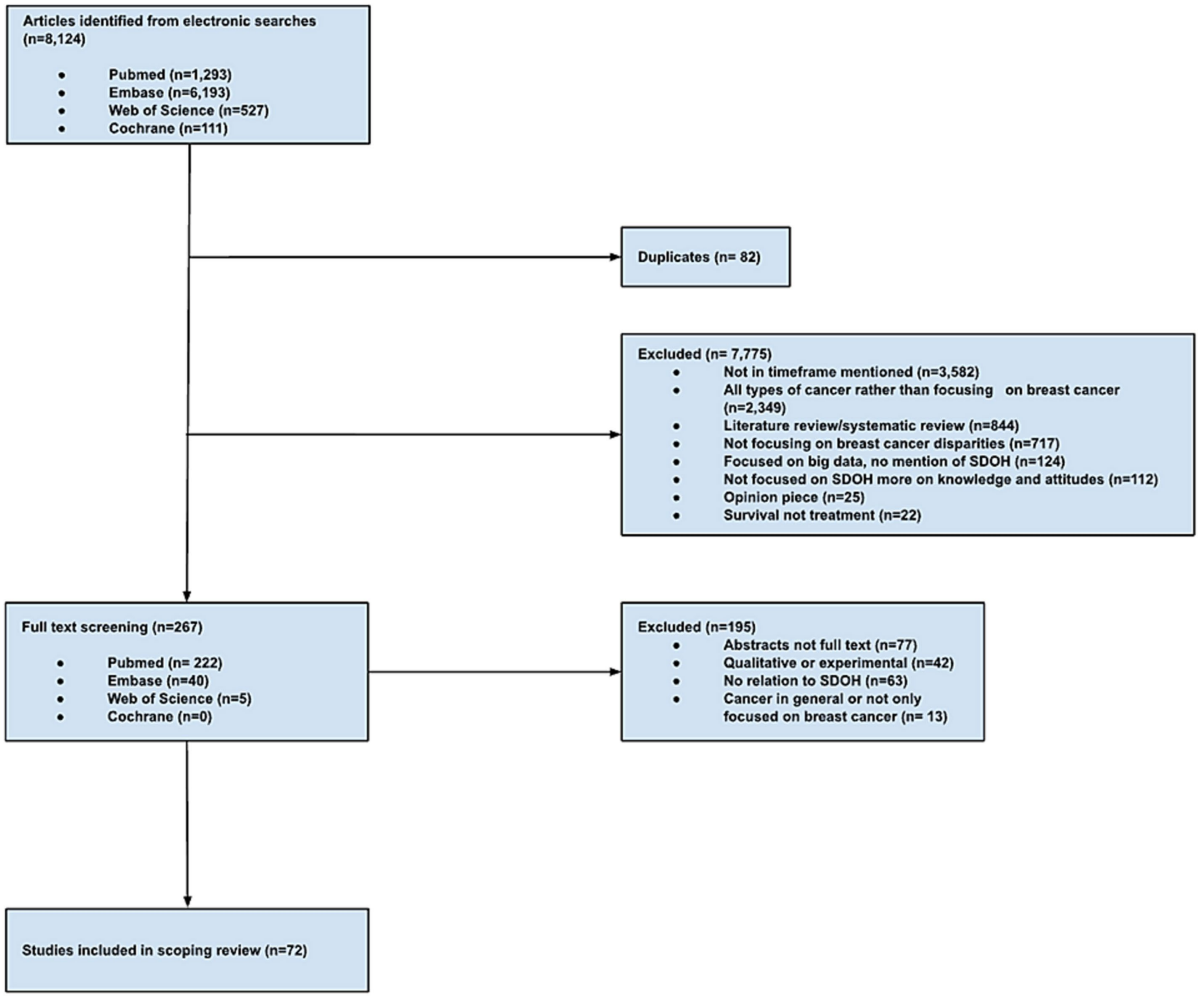
PRISMA-ScR flow chart of study selection process.

The initial study extraction resulted in 8,124 articles from PubMed (*n* = 1,293), EMBASE (*n* = 6,193), Web of Science (*n* = 527), and Cochrane (*n* = 111). Studies were excluded due to publication outside of the timeframe (*n* = 7,775), discussion of all types of cancer rather than focusing on breast cancer (*n* = 2,349), being a literature review or systematic review (*n* = 884), lack of focus on breast cancer disparities (*n* = 717), focusing on big data or no mention of SDOH (*n* = 124), focusing more on knowledge and attitudes rather than SDOH (*n* = 112), being an opinion piece or an editorial (*n* = 25), or emphasizing survival as an outcome rather than treatment (*n* = 22). Duplicate studies were also excluded (*n* = 82 from PubMed, *n* = 60 from EMBASE, *n* = 20 from Web of Science, and *n* = 2 from Cochrane). A total of 267 studies met the inclusion criteria from PubMed (*n* = 222), EMBASE (*n* = 40), and Web of Science (*n* = 5). An additional 195 studies were excluded after a full study review due to being an abstract and not a full text (*n* = 77), having a qualitative or experimental study design (*n* = 42), having no relation to SDOH (*n* = 63), and discussing cancer types in general rather than narrowing it down to breast cancer (*n* = 13). A total of 72 studies were retained for analysis (Figure 1).

The 72 included studies were published between 2013 and 2023. About half of the studies (58%) were published in 2018 or later (*n* = 42). Study designs included cross-sectional studies (*n* = 45); cohort studies (*n* = 18); and case–control studies (*n* = 9). Sample size ranged from *n* = 100 to *n* = 3,821,084 female adults with breast cancer while the age of this target population ranged from 40 to 89 years old (Table 1).

### Priority populations

Priority populations who were actively involved (or targeted) in implementation activities were ethnically diverse female patients diagnosed with breast cancer including African American women; Muslim and Christian Arab American; Haitian women; Filipino women; and Korean American women. Another set of studies focused on women from programs, such as women from Geisel School of Medicine (*n* = 3,413), from the BSPAN program (*n* = 19,292), women who underwent mammography in Harvard Medical School (*n* = 9,575), female patients from a single institution undergoing breast radiotherapy (*n* = 1,057), presenting to radiology department (*n* = 758), mammogram facilities (*n* = 1,749), and at a quaternary care academic medical center (*n* = 738) (Table 1).

Additional studies focused on the characteristics of the women, such as women who have individual subscribers or employer supplemented (*n* = 95,661), are Medicaid-insured and Medicare fee-for service (*n* = 11), are insured but have not undergone mammogram in 24 months (*n* = 47,946), have no history of breast cancer (*n* = 181,755), have telephone access (*n* = 169,116), homeless women (*n* = 100), hospitalized women (*n* = 250), are medically underserved (*n* = 518), and have limited accessibility to mammogram (*n* = 73,718) (Table 1).

### Classification of SDOH factors influencing breast cancer screening behavior based on the healthy people 2030 categories

An examination of SDOH influential factors of breast cancer screening was conducted, focusing on their classification into Healthy People 2030 categories (20). Among the various SDOH identified, those related to socioeconomic status (*n* = 96) exhibited the highest frequency (Table 1). Specifically, factors such as income (*n* = 32), education level (*n* = 29), employment status (*n* = 8), birthplace/citizenship (*n* = 5), acculturation/years lived in the United States (*n* = 5), marital status (*n* = 2), social support (*n* = 2), and number of children (*n* = 1) were among the key elements. Access to healthcare (*n* = 75) emerged as a significant theme, with subcategories like insurance status (*n* = 33), accessibility of healthcare services and providers (*n* = 18), insurance coverage (*n* = 8), access to mammography facilities (*n* = 5), insurance copayments (*n* = 2), time from breast cancer diagnosis to first treatment (*n* = 1), travel time to clinic (*n* = 1), and county uninsured rate (*n* = 1) also being identified. Race/Ethnicity (*n* = 79), age (*n* = 52), sex/gender (*n* = 2), and sexual orientation (*n* = 1) were additional factors reported. Language-related SDOH (*n* = 21) were observed 21 times, encompassing language proficiency/preferred language (*n* = 15) and health literacy (*n* = 6). Furthermore, location (*n* = 30), transportation (*n* = 5), housing (*n* = 3), county poverty rate (*n* = 2), internet access (*n* = 1), area deprivation index (*n* = 1), diversity index (*n* = 1), cultural and religious beliefs (*n* = 4), perceived discrimination (*n* = 2), health beliefs (*n* = 1), and trust in health care providers/systems (*n* = 1) were also cited. Finally, health-related factors (*n* = 9) that were reported include comorbidities and chronic illnesses (*n* = 3), BMI (*n* = 2), medical/family history of breast cancer (*n* = 1), history of mental illness (*n* = 1), HIV status (*n* = 1), and substance/alcohol abuse (*n* = 1) (Table 1). Among the Healthy People 2030 categories, Social and Community Context (*n* = 177) emerged as the most prevalent, with a striking 177 occurrences of SDOH. Following closely behind were Healthcare Access and Quality (*n* = 80), Economic Stability (*n* = 56), Neighborhood and Built Environment (*n* = 46), and Education Access and Quality (*n* = 36) (Table 1).

### Database access and characteristics

Databases with the highest number of occurrences include data from the National Health Interview Survey (*n* = 8) [over a range of years from 2005 to 2018], the Breast Cancer Surveillance Consortium (*n* = 4), and the United States Department of Health (*n* = 2). Other databases used include the National Program of Cancer Registries, the National Assessment of Adult Literacy, and SEER Medicare. Of the 74 databases used, 47% (*n* = 35) are publicly available. The databases are available at the city (*n* = 16), county (*n* = 1), state (*n* = 28), and national (*n* = 30) levels (Table 2).

### Significance of association between SDOH factors and access to mammography and treatment opportunities

The Health Care Access and Quality category was reported in the highest number of studies (*n* = 44; 61%), showing its statistical significance in relation to access to mammography. Insurance status was the most reported sub-categorical factor of Health Care Access and Quality with *n* = 36 (50%) articles supporting this finding. A total of *n* = 42 (58%) studies showed statistical significance in the social and community context category, with the highest subcategories being age and ethnicity with *n* = 46 (63%) and *n* = 40 (55%) articles denoting their significance, respectively. Language was the third highest with *n* = 11 (15%) studies highlighting its significance as an influential factor of screening behavior. Further, *n* = 28 (38%) studies exhibited statistical significance under the Economic Stability category, with income level being the most common sub-categorical indicator emphasized in *n* = 20 (27%) studies. Next, the Neighborhood and Built Environment category showed statistical significance in *n* = 18 (25%) articles, with zip code or geographic location being reported as the strongest sub-categorical indicator in *n* = 15 studies (20%). Moreover, *n* = 24 (33%) articles showed statistical significance in Education Access and Quality as strong indicators of mammography rate, with the highest level of education completed acting as the strongest sub-categorical factor in *n* = 24 (33%) articles (Table 1).

The methodology used across the included studies to communicate statistical data were reported as: logistic regression (*n* = 63), descriptive statistics (*n* = 23), chi-square tests (*n* = 20), T-tests (*n* = 13), linear regression (*n* = 9), multivariate analyses (*n* = 9), Wald tests (*n* = 8), Generalized estimating equations (*n* = 7), Spatial analysis (*n* = 7), Cox proportional hazards regression (*n* = 5), Kaplan–Meier cumulative incidence (*n* = 3), Sensitivity analysis (*n* = 2), Trend analysis (*n* = 2), and *Z* tests (*n* = 1) (Table 1).

### Lessons learned

Using the three phases of qualitative content analysis delineated by Elo and Kyngas (19), qualitative themes were identified. First, data relevant to lessons learned was collected from each of the included studies in the preparation stage (Phase I) (Supplementary material 1). Second, lessons learned were organized into bullet points and tabulated by primary author to compare data across studies and explore emerging themes (Phase 2) (Supplementary material 1). Major themes were then highlighted in Table 3 (Phase III).

Many of the studies demonstrated a strong association between a lack of health insurance and a lower rate of breast cancer screening (21–25). Ethnic minority women, with the exception of those identifying as Asian, had a lower likelihood of being screened, and Black women experienced a higher risk of diagnosis upon first screening (25–29). While few studies analyze the effect of sexual orientation on breast cancer screening, initial insights reveal there are significant differences in mammography between bisexual, lesbian, and heterosexual women regardless of racial/ethnic groups (30). In considering religious values, fatalism-emphasizing religions were associated with less screening adherences and maintenance of modesty did not prove a significant limitation for women receiving mammograms (31–33). Economic factors present limitations as both high levels of poverty and impoverished rural regions were associated with lower screening rates (27, 32, 34–37). Improving patient-provider communication, addressing perceived discrimination, and improving trust in the health care system is necessary to improve screening rates across all demographics (38–42). Additionally, structural efforts to improve health insurance coverage, language proficiency, and transportation services could be beneficial (20–110). These steps will need to involve the local community to develop community-tailored educational campaigns to reinforce the importance of establishing yearly mammogram screenings (Table 3) (22, 34, 46, 49, 54, 55, 70, 76, 80, 86).

## Discussion

The purpose of this scoping review was to identify the major SDOH acting as influential factors of breast cancer screening in United States women aged ≥ 40 years old. The analysis of the 72 included studies can inform which SDOH categories to focus on when designing evidence-based interventions for more effective and sustained positive behavior and health outcomes among United States women at-risk of breast cancer.

### SDOH factors and healthy people 2030 categories

Of the classifications of SDOH by Healthy People 2030, the Social and Community Context Category was the most prevalent across the included studies (*n* = 177). However, when looking at the most frequently cited SDOH influential factors of breast cancer screening behaviors, those related to socioeconomic status exhibited the highest frequency. Such factors included income (*n* = 32), education level (*n* = 29), employment status (*n* = 8), birthplace/citizenship (*n* = 5), acculturation/years lived in the United States (*n* = 5), marital status (*n* = 2), social support (*n* = 2), and number of children (*n* = 1). Other highly reported factors include insurance status (*n* = 33) under the Healthcare Access and Quality category, as well as race/ethnicity (*n* = 79) and age (*n* = 52) under the Social and Community Context Category.

There is evidence to show the significance of the relationship between socioeconomic factors and breast cancer screening. Over 30 different interventions that address SDOH increased breast cancer screening rates by 12.3% (93). Social determinants such as poverty, lack of education, neighborhood disadvantage, residential segregation, racial discrimination, lack of social support, and social isolation have shown in numerous studies to play a role in the breast cancer stage at diagnosis (94, 95). Gomez et al. (94) highlighted in their review that social and built environments have been shown to factor into cancer diagnoses in 82% of 34 reviewed articles published since 2010, including breast cancer (96). Studies have found that, not only do these factors have a significant association with breast cancer screening individually, but they also work dynamically to impact screening and treatment for breast cancer (97).

Low affordability and healthcare accessibility profoundly impact breast cancer screening, leading to lower adherence in female patients. For instance, Medicaid patients who are required to pay co-payments for preventative services as well as for recommended follow-up visits are less likely to pursue such preventative services and mammograms are included in lost care (96). Co-payments of more than $10 have been associated with reduced rates of mammograms (97). Furthermore, a study investigating breast cancer screening among young military women revealed that, when removing cost and access barriers to obtaining a breast mammography, first-time screening rates were 90% (98). Similar results have been noted when patients were provided free mammograms in underserved areas. The Building Relationships and Initiatives Dedicated to Gaining Equality (BRIDGE) Healthcare Clinic, a free clinic offered by the University of South Florida, provided patients free mammograms and noted that about 84.5% of patients utilized these services (99).

### Significance of associations between SDOH factors and breast cancer screening and treatment

The majority of the studies reported a significant association between the SDOH factors under each of the five Healthy People 2030 categories. Insurance status was the most reported sub-categorical factor of Health Care Access and Quality with *n* = 36 (50%) articles supporting this finding. Insurance status often determines whether patients seek mammography services as they often become costly without robust coverage (93). Despite stable mammography rates among women in the United States between the years 2000 and 2015, women who report being uninsured consistently have the lowest rates of mammography at 35.3% (100).

Moreover, a total of *n* = 42 (58%) studies showed statistical significance in the social and community context category, with the highest subcategories being age and ethnicity with *n* = 46 (63%) and *n* = 40 (55%) articles denoting their significance, respectively. Health disparities in the United States have been consistently associated with delayed screening, which then contributes to higher mortality rates among both Hispanic and Black populations (28). Inequities also exist in mammography rates between patients of different sexual orientations (111). White, bisexual women had significantly lower mammography rates than White, heterosexual women, while mammography rates were significantly higher for bisexual, Black women than for heterosexual, Black women (102).

Income (*n* = 20; 27%) strongly influences mammography rates since women with estimated household incomes greater than $38,100 have been found to have rates of repeat mammography higher than those of women below $25,399 (109). In addition to household income, food security acts as another influential factor of mammography rates. When patients are forced to choose between feeding their families and pursuing preventative care, mammography becomes more of a luxury than lifesaving care (110). Women facing food insecurity have shown a 54% lower likelihood of obtaining mammography (110).

Language (*n* = 11; 15%) and availability of translation services, health literacy, and culture also play a strong role in mammography rates since many women with limited English proficiency seek mammography care and receive abnormal results (103). Appropriate, timely follow-up in the correct language is imperative to proper care provision; however, a lack of translation services worsens the language barrier between these patients and their healthcare providers, delaying care (101). Clinics with a patient population that is majority non-English speaking also experience greater follow-up delays than those with a minority of non-English speakers due to language barriers (103). The lower a patient’s health literacy, the less likely they are to undergo up-to-date breast cancer screening according to official guidelines (104, 105). The cultural and religious beliefs in fatalism have also been continuously found to be associated with lower mammography rates, whereby women with the highest beliefs in fatalism had the lowest breast cancer screening rates (106, 107).

Finally, Education Access and Quality sub-categories were significant indicators of mammography rate, with the highest level of education completed acting as the strongest sub-categorical factor in *n* = 24 (33%) articles. A systematic review by Damiani et al. (109) showed that United States women with the highest level of education were more likely to screen for breast cancer, with a 36% higher rate of adherence to national screening guidelines compared to women with lower levels of education. This finding holds health professionals and community outreach efforts accountable in ensuring that the local patient population is aware of the importance of and has access to breast cancer screening measures (109, 110).

### Availability of public databases

Of the 74 databases used, only 47% (*n* = 35) were publicly available. There is a need to establish more widely accessible databases encompassing a routine collection of data on the SDOH to allow for the examination of additional evidence on exiting associations between SDOH and health outcomes. These databases could also inform the development and implementation of longitudinal and experimental studies at the county, city, and national levels to decrease health disparities exacerbated by SDOH factors.

### Strengths and limitations

Despite the importance of this study in guiding and informing the development and implementation of future SDOH-oriented evidence-based interventions for breast cancer screening, findings need to take into consideration this study’s limitations. First, despite a comprehensive search of the literature in psychosocial databases compatible with the topic at hand, this review did not include gray literature and did not encompass tracing of reference lists in included studies. Second, it also was limited to observational studies to explore SDOH factors acting as factors based on statistical tests looking at significance of reported associations. These observational studies also widely varied in reported sample sizes, ranging from 100 participants to a population of 4 million. Therefore, although statistical significance was reported across different studies, effect sizes, power, and external validity varied greatly. Future systematic reviews should assess the rigor and quality of analysis carried out, evaluate recruitment efforts and data collection methods, and critique analytical tests carried out to account for the difference in sample sizes. Third, the mesh terms included as many technical words and keywords relevant to the SDOH as possible but might have inadvertently omitted some key words due to the continuously evolving and changing definitions related to SDOH. However, the help of an expert research librarian mitigated the impact of this concern by imposing rigor in implemented scoping review protocols when developing the search strategy for this review. Fourth, formal assessment of the methodology and quality of the evidence was beyond the scope of this study and relied on the reported statistical tests to assess significance. Follow-up systematic reviews would help with addressing this limitation by focusing specifically on the analytical proportion of each study. Fifth, although various categorizations exist for SDOH such as the WHO and CDC categories, the Healthy People 2030 taxonomy was adopted for use as it is the most recently updated classification encompassing a wide range of SDOH. Future studies should compare these taxonomies by feasibility, usability, and importance for a more valid and systematic approach to SDOH categorization.

## Conclusion

This scoping review describes major SDOH acting as significant influential factors of breast cancer screening behaviors among United States women aged ≥40 years old who are at-risk of the disease. Results may inform future evidence-based interventions aiming to address the underlying factors contributing to low screening rates for breast cancer in the United States. Efforts to integrate SDOH within the different components of intervention planning, implementation, and sustainability are widely gaining recognition, particularly in underserved communities, due to their substantial influence on everyday behaviors.

## Data availability statement

The original contributions presented in the study are included in the article/Supplementary material, further inquiries can be directed to the corresponding author.

## Author contributions

VJ: Conceptualization, Data curation, Methodology, Writing – original draft. DL: Conceptualization, Data curation, Methodology, Writing – original draft. GO: Conceptualization, Data curation, Methodology, Writing – original draft. YZ: Conceptualization, Data curation, Methodology, Writing – original draft. SB: Conceptualization, Data curation, Methodology, Writing – original draft. AM: Conceptualization, Data curation, Methodology, Writing – original draft. SD: Conceptualization, Data curation, Methodology, Writing – original draft. MR: Conceptualization, Data curation, Methodology, Writing – original draft. DD: Methodology, Writing – original draft. MK: Methodology, Software, Writing – review & editing. LS: Conceptualization, Investigation, Methodology, Project administration, Supervision, Validation, Writing – review & editing.

## References

[1] World Health Organization (2014). World conference on social determinants of health: case studies on social determinants. Available at: http://www.who.int/sdhconference/resources/case_studies/en/

[2] Office of Disease Prevention and Health Promotion (n.d.). Social determinants of health. Healthy people 2030. U.S. Department of Health and Human Services. Available at: https://health.gov/healthypeople/priority-areas/social-determinants-health

[3] Krause TM Schaefer C Highfield L. The association of social determinants of health with health outcomes. Am J Manag Care. (2021) 27:e89–96. doi: 10.37765/ajmc.2021.88603, PMID: 33720674

[4] Agency for Healthcare Research and Quality (2021). 2021 National Healthcare Quality and Disparities Report: Executive Summary. 35263063

[5] Liu D Schuchard H Burston B Yamashita T Albert S. Interventions to reduce healthcare disparities in cancer screening among minority adults: a systematic review. J Racial Ethn Health Disparities. (2021) 8:107–26. doi: 10.1007/s40615-020-00763-1, PMID: 32415578

[6] Zavala VA Bracci PM Carethers JM Carvajal-Carmona L Coggins NB Cruz-Correa MR . Cancer health disparities in racial/ethnic minorities in the United States. Br J Cancer. (2021) 124:315–32. doi: 10.1038/s41416-020-01038-6, PMID: 32901135 PMC7852513

[7] Ward E Jemal A Cokkinides V Singh GK Cardinez C Ghafoor A . Cancer disparities by race/ethnicity and socioeconomic status. CA Cancer J Clin. (2004) 54:78–93. doi: 10.3322/canjclin.54.2.78 15061598

[8] Mathers C Loncar D. Projections of global mortality and burden of disease from 2002 to 2030. PLoS Med. (2006) 3:e442. doi: 10.1371/journal.pmed.0030442, PMID: 17132052 PMC1664601

[9] U.S. Preventive Services Task Force (2016). Breast cancer screening final recommendations. Available at: http://screeningforbreastcancer.org/ (Accessed May 26, 2023).

[10] Coughlin SS Uhler RJ Richards T Wilson KM. Breast and cervical cancer screening practices among Hispanic and non-Hispanic women residing near the United States-Mexico border, 1999–2000. Fam Commun Health. (2003) 26:130–9. doi: 10.1097/00003727-200304000-00006, PMID: 12802118

[11] Jones AR Caplan LS Davis MK. Racial/ethnic differences in the self-reported use of screening mammography. J Community Health. (2003) 28:301–16. doi: 10.1023/a:1025451412007 14535597

[12] Rodriguez MA Ward LM Perez-Stable EJ. Breast and cervical cancer screening: impact of health insurance status, ethnicity, and nativity of Latinas. Ann Fam Med. (2005) 3:235–41. doi: 10.1370/afm.291, PMID: 15928227 PMC1466881

[13] Lantz PM Mujahid M Schwartz K Janz NK Fagerlin A Salem B . The influence of race, ethnicity, and individual socioeconomic factors on breast cancer stage at diagnosis. Am J Public Health. (2006) 96:2173–8. doi: 10.2105/AJPH.2005.072132, PMID: 17077391 PMC1698157

[14] Abraido-Lanza AF Chao MT Gammon MD. Breast and cervical cancer screening among Latinas and non-Latina whites. Am J Public Health. (2004) 94:1393–8. doi: 10.2105/AJPH.94.8.1393, PMID: 15284049 PMC1448461

[15] Tricco AC Lillie E Zarin W O’Brien KK Colquhoun H Levac D . PRISMA extension for scoping reviews (PRISMA-ScR): checklist and explanation. Ann Intern Med. (2018) 169:467–73. doi: 10.7326/M18-0850, PMID: 30178033

[16] Arksey H O’Malley L. Scoping studies: towards a methodological framework. Int J Soc Res Methodol. (2005) 8:19–32. doi: 10.1080/1364557032000119616

[17] American Cancer Society. ACS breast cancer screening guidelines. Cancer.org. (2023). Available at: https://www.cancer.org/cancer/types/breast-cancer/screening-tests-and-early-detection/american-cancer-society-recommendations-for-the-early-detection-of-breast-cancer.html (Accessed January 16, 2024).

[18] Office of Disease Prevention and Health Promotion (2023). Social determinants of health. Health.gov. Available at: https://health.gov/healthypeople/objectives-and-data/social-determinants-health (Accessed August 15, 2023).

[19] Elo S Kyngäs H. The qualitative content analysis process. J Adv Nurs. (2008) 62:107–15. doi: 10.1111/j.1365-2648.2007.04569.x 18352969

[20] Henderson LM O’Meara ES Haas JS Lee CI Kerlikowske K Sprague BL . The role of social determinants of health in self-reported access to health care among women undergoing screening mammography. J Women's Health. (2020) 29:1437–46. doi: 10.1089/jwh.2019.8267, PMID: 32366199 PMC7703148

[21] Beaber EF Tosteson ANA Haas JS Onega T Sprague BL Weaver DL . Breast cancer screening initiation after turning 40 years of age within the PROSPR consortium. Breast Cancer Res Treat. (2016) 160:323–31. doi: 10.1007/s10549-016-3990-x, PMID: 27665586 PMC5576986

[22] Jin SW Lee HY Lee J. Analyzing factors of breast cancer screening adherence among Korean American women using Andersen’s behavioral model of healthcare services utilization. Ethn Dis. (2019) 29:427–34. doi: 10.18865/ed.29.s2.427, PMID: 31308615 PMC6604780

[23] Kosog K Earle M Stellon E Nolan C Wainwright MK Webb T . Identifying an association between socio-demographic factors and breast cancer screening adherence in a federally qualified health Centre sample in the United States. A retrospective, cross-sectional study. Health Soc Care Commun. (2020) 28:1772–9. 10.1111/hsc.13002 32304270

[24] Kempe KL Larson RS Shetterley S Wilkinson A. Breast cancer screening in an insured population: whom are we missing? Perm J. (2013) 17:38–44. doi: 10.7812/TPP/12-068, PMID: 23596367 PMC3627794

[25] Selove R Kilbourne B Fadden MK Sanderson M Foster M Offodile R . Time from screening mammography to biopsy and from biopsy to breast cancer treatment among black and white, women medicare beneficiaries not participating in a health maintenance organization. Womens Health Issues. (2016) 26:642–7. doi: 10.1016/j.whi.2016.09.003, PMID: 27773529 PMC5116399

[26] Wilcox ML Acuña JM Ward-Peterson M Alzayed A Alghamdi M Aldaham S. Racial/ethnic disparities in annual mammogram compliance among households in little Haiti, Miami-Dade County, Florida: an observational study. Medicine. (2016) 95:e3826. doi: 10.1097/md.0000000000003826, PMID: 27399061 PMC5058790

[27] Wilkerson AD Obi M Ortega C Sebikali-Potts A Wei W Pederson HJ . Young black women may be more likely to have first mammogram cancers: a new perspective in breast cancer disparities. Ann Surg Oncol. (2023) 30:2856–69. doi: 10.1245/s10434-022-12995-y, PMID: 36602665

[28] Agénor M Pérez AE Tabaac AR Bond KT Charlton BM Bowen DJ . Sexual orientation identity disparities in mammography among White, black, and Latina US women. LGBT Health. (2020) 7:312–20. doi: 10.1089/lgbt.2020.0039, PMID: 32668184 PMC7475089

[29] Alatrash M. Determinants of breast cancer screening in three Arab American women subgroups. J Transcult Nurs. (2021) 32:749–56. doi: 10.1177/10436596211008215 33855910

[30] Jensen B Khan H Layeequr RR. Sociodemographic determinants in breast cancer screening among uninsured women of West Texas. Medicina. (2022) 58:1–12. doi: 10.3390/medicina58081010, PMID: 36013477 PMC9416323

[31] Anderson RT Yang T-C Matthews SA Camacho F Kern T Mackley HB . Breast cancer screening, area deprivation, and later-stage breast cancer in Appalachia: does geography matter? Health Serv Res. (2014) 49:546–67. doi: 10.1111/1475-6773.12108, PMID: 24117371 PMC3976186

[32] Hong HC Ferrans CE Park C Lee H Quinn L Collins EG. Effects of perceived discrimination and trust on breast cancer screening among Korean American women. Womens Health Issues. (2018) 28:188–96. doi: 10.1016/j.whi.2017.11.001, PMID: 29223326

[33] Agrawal P Chen TA McNeill LH Acquati C Connors SK Nitturi V . Factors associated with breast cancer screening adherence among church-going African American women. Int J Environ Res Public Health. (2021) 18:8494. doi: 10.3390/ijerph18168494, PMID: 34444241 PMC8392666

[34] Onega T Tosteson TD Weiss J Haas JS Goodrich M DiFlorio R . Multi-level influences on breast cancer screening in primary care. J Gen Intern Med. (2018) 33:1729–37. doi: 10.1007/s11606-018-4560-1, PMID: 30076569 PMC6153219

[35] Dong W Rose J Kim U Cooper GS Tsui J Koroukian SM. Medicaid expansion associated with reduction in geospatial breast cancer stage at diagnosis disparities. J Public Health Manag Pract. (2022) 28:469–77. doi: 10.1097/PHH.0000000000001514, PMID: 35420579 PMC9308621

[36] Henderson LM Benefield T Nyante SJ Marsh MW Greenwood-Hickman MA Schroeder BF. Performance of digital screening mammography in a population-based cohort of black and white women. Cancer Causes Control. (2015) 26:1495–9. doi: 10.1007/s10552-015-0631-3, PMID: 26184718 PMC4567941

[37] Patel K Kanu M Liu J Bond B Brown E Williams E . Factors influencing breast cancer screening in low-income African Americans in Tennessee. J Community Health. (2014) 39:943–50. doi: 10.1007/s10900-014-9834-x, PMID: 24554393 PMC4165808

[38] Johnson CJ Morawski BM Hobbs L Lewis D Cariou C Rycroft RK. Time from breast cancer diagnosis to treatment among Idaho’s National Breast and cervical Cancer early detection program population, 2011-2017. Cancer Causes Control. (2021) 32:667–73. doi: 10.1007/s10552-021-01407-3, PMID: 33665701

[39] Khaliq W Aamar A Wright SM. Predictors of non-adherence to breast cancer screening among hospitalized women. PLoS One. (2015) 10:e0145492. doi: 10.1371/journal.pone.0145492, PMID: 26709510 PMC4692526

[40] Lee CI Bogart A Germino JC Goldman LE Hubbard RA Haas JS . Availability of advanced breast imaging at screening facilities serving vulnerable populations. J Med Screen. (2016) 23:24–30. doi: 10.1177/0969141315591616, PMID: 26078275 PMC4679713

[41] Virk-Baker MK Martin MY Levine RS Wang X Nagy TR Pisu M. Mammography utilization among black and White Medicare beneficiaries in high breast cancer mortality US counties. Cancer Causes Control. (2013) 24:2187–96. doi: 10.1007/s10552-013-0295-9, PMID: 24077760 PMC3955601

[42] Wiese D Islami F Henry KA. Changes in geographic accessibility to mammography by state and rural-urban status, United States, 2006-2022. J Natl Cancer Inst. (2023) 115:337–40. doi: 10.1093/jnci/djac217, PMID: 36515214 PMC9996203

[43] Oviedo AD. Mammogram adherence among Filipino American women. J Immigr Minor Health. (2022) 24:639–44. doi: 10.1007/s10903-021-01223-6, PMID: 34089445

[44] Nair RG Lee SJC Berry E Argenbright KE Tiro JA Skinner CS. Long-term mammography adherence among uninsured women enrolled in the breast screening and patient navigation (BSPAN) program. Cancer Epidemiol Biomarkers Prev. (2022) 31:77–84. doi: 10.1158/1055-9965.EPI-21-0191, PMID: 34750203 PMC8755604

[45] Alabdullatif N Arrieta A Dlugasch L Hu N. The impact of IT-based healthcare communication on mammography screening utilization among women in the United States: National Health Interview Survey (2011-2018). Int J Environ Res Public Health. (2022) 19:12737. doi: 10.3390/ijerph191912737, PMID: 36232036 PMC9566602

[46] Asgary R Garland V Sckell B. Breast cancer screening among homeless women of new York City shelter-based clinics. Womens Health Issues. (2014) 24:529–34. doi: 10.1016/j.whi.2014.06.002, PMID: 25029909

[47] Ayanian JZ Landon BE Zaslavsky AM Newhouse JP. Racial and ethnic differences in use of mammography between Medicare advantage and traditional Medicare. J Natl Cancer Inst. (2013) 105:1891–6. doi: 10.1093/jnci/djt333, PMID: 24316600 PMC3866158

[48] Balazy KE Benitez CM Gutkin PM Jacobson CE von Eyben R Horst KC. Association between primary language, a lack of mammographic screening, and later stage breast cancer presentation. Cancer. (2019) 125:2057–65. doi: 10.1002/cncr.32027, PMID: 30768784

[49] Beaber EF Sprague BL Tosteson ANA Haas JS Onega T Schapira MM . Multilevel predictors of continued adherence to breast cancer screening among women ages 50-74 years in a screening population. J Women's Health. (2019) 28:1051–9. doi: 10.1089/jwh.2018.6997, PMID: 30481098 PMC6703243

[50] Calo WA Vernon SW Lairson DR Linder SH. Area-level socioeconomic inequalities in the use of mammography screening: a multilevel analysis of the health of Houston survey. Womens Health Issues. (2016) 26:201–7. doi: 10.1016/j.whi.2015.11.002, PMID: 26809487 PMC4761271

[51] Castañeda SF Malcarne VL Foster-Fishman PG Davidson WS Mumman MK Riley N . Health care access and breast cancer screening among Latinas along the California-Mexican border. J Immigr Minor Health. (2014) 16:670–81. doi: 10.1007/s10903-013-9938-x, PMID: 24150421 PMC4076386

[52] Cataneo JL Meidl H Ore AS Raicu A Schwarzova K Cruz CG. The impact of limited language proficiency in screening for breast cancer. Clin Breast Cancer. (2023) 23:181–8. doi: 10.1016/j.clbc.2022.11.008, PMID: 36635166

[53] Chandak A Nayar P Lin G. Rural-urban disparities in access to breast cancer screening: a spatial clustering analysis: disparities in breast cancer screening. J Rural Health. (2019) 35:229–35. doi: 10.1111/jrh.12308, PMID: 29888497

[54] Christensen EW Pelzl CE Patel BK Carlos RC Rula EY. Urbanicity, income, and mammography-use disparities among American Indian women. Am J Prev Med. (2023) 64:611–20. doi: 10.1016/j.amepre.2023.01.013, PMID: 37085244

[55] Clark CR Tosteson TD Tosteson ANA Onega T Weiss JE Harris KA . Diffusion of digital breast tomosynthesis among women in primary care: associations with insurance type. Cancer Med. (2017) 6:1102–7. doi: 10.1002/cam4.1036, PMID: 28378409 PMC5430135

[56] Clarke TC Endeshaw M Duran D Saraiya M. Breast cancer screening among women by nativity, birthplace, and length of time in the United States. Natl Health Stat Rep. (2019) 129:1–15. 31751203

[57] Davis J Liang J Petterson MB Roh AT Chundu N Kang P . Risk factors for late screening mammography. Curr Probl Diagn Radiol. (2019) 48:40–4. doi: 10.1067/j.cpradiol.2017.10.014 29273558

[58] Duggan C Molina Y Carosso E Ibarra G Thompson B. County of residence and screening practices among Latinas and non-Latina whites in two rural communities. Ethn Dis. (2019) 29:31–8. doi: 10.18865/ed.29.1.31, PMID: 30713414 PMC6343545

[59] Elkin EB Paige Nobles J Pinheiro LC Atoria CL Schrag D. Changes in access to screening mammography, 2008-2011. Cancer Causes Control. (2013) 24:1057–9. doi: 10.1007/s10552-013-0180-6 23468282 PMC3647343

[60] Fedewa SA de Moor JS Ward EM DeSantis CE Goding Sauer A Smith RA . Mammography use and physician recommendation after the 2009 U.S. preventive services task force breast cancer screening recommendations. Am J Prev Med. (2016) 50:e123–31. doi: 10.1016/j.amepre.2015.10.010, PMID: 26699245

[61] Flores EJ López D Miles RC Glover M 4th Lehman CD Harvey HB . Impact of primary care physician interaction on longitudinal adherence to screening mammography across different racial/ethnic groups. J Am Coll Radiol. (2019) 16:908–14. doi: 10.1016/j.jacr.2018.12.020, PMID: 30737162

[62] Guo Y Cheng TC Yun LH. Factors associated with adherence to preventive breast cancer screenings among middle-aged African American women. Soc Work Public Health. (2019) 34:646–56. doi: 10.1080/19371918.2019.1649226, PMID: 31411130

[63] Henry KA McDonald K Sherman R Kinney AY Stroup AM. Association between individual and geographic factors and nonadherence to mammography screening guidelines. J Women's Health. (2014) 23:664–74. doi: 10.1089/jwh.2013.4668, PMID: 24865409 PMC4129969

[64] Hubbard RA O’Meara ES Henderson LM Hill D Braithwaite D Haas JS . Multilevel factors associated with long-term adherence to screening mammography in older women in the U.S. Prev Med. (2016) 89:169–77. doi: 10.1016/j.ypmed.2016.05.034, PMID: 27261409 PMC4969188

[65] Jena AB Huang J Fireman B Fung V Gazelle S Landrum MB . Screening mammography for free: impact of eliminating cost sharing on cancer screening rates. Health Serv Res. (2017) 52:191–206. doi: 10.1111/1475-6773.12486, PMID: 26990550 PMC5264125

[66] Kim E Moy L Gao Y Hartwell CA Babb JS Heller SL. City patterns of screening mammography uptake and disparity across the United States. Radiology. (2019) 293:151–7. doi: 10.1148/radiol.2019190647, PMID: 31429681

[67] Kim SE Bachorik AE Bertrand KA Gunn CM. Differences in breast cancer screening practices by diabetes status and race/ethnicity in the United States. J Women's Health. (2022) 31:848–55. doi: 10.1089/jwh.2021.0396, PMID: 34935471 PMC9347336

[68] Komenaka IK Nodora JN Hsu C-H Martinez ME Gandhi SG Bouton ME . Association of health literacy with adherence to screening mammography guidelines. Obstet Gynecol. (2015) 125:852–9. doi: 10.1097/aog.0000000000000708, PMID: 25751204

[69] Lapeyrouse LM Miranda PY Morera OF Heyman JM Balcazar HG. Healthcare use and mammography among Latinas with and without health insurance near the US-Mexico border. J Racial Ethn Health Disparities. (2017) 4:282–7. doi: 10.1007/s40615-016-0227-y, PMID: 27072542

[70] Lee HY Lee MH Jang YJ Lee DK. Breast cancer screening disparity among Korean American immigrant women in Midwest. Asian Pac J Cancer Prev. (2017) 18:2663–7. doi: 10.22034/APJCP.2017.18.10.2663, PMID: 29072066 PMC5747386

[71] Lee CI Zhu W Onega T Henderson LM Kerlikowske K Sprague BL . Comparative access to and use of digital breast tomosynthesis screening by women’s race/ethnicity and socioeconomic status. JAMA Netw Open. (2021) 4:e2037546. doi: 10.1001/jamanetworkopen.2020.37546 33606032 PMC7896194

[72] Li L Ji J Besculides M Bickell N Margolies LR Jandorf L . Factors associated with mammography use: a side-by-side comparison of results from two national surveys. Cancer Med. (2020) 9:6430–51. doi: 10.1002/cam4.3128, PMID: 32677744 PMC7476827

[73] Luo Y Carretta H Lee I LeBlanc G Sinha D Rust G. Naïve Bayesian network-based contribution analysis of tumor biology and healthcare factors to racial disparity in breast cancer stage-at-diagnosis. Health Inf Sci Syst. (2021) 9:35. doi: 10.1007/s13755-021-00165-5, PMID: 34631040 PMC8463645

[74] Molina Y Plascak JJ Patrick DL Bishop S Coronado GD Beresford SAA. Neighborhood predictors of mammography barriers among US-based Latinas. J Racial Ethn Health Disparities. (2017) 4:233–42. doi: 10.1007/s40615-016-0222-3, PMID: 27059049 PMC5055845

[75] Monsivais P Amiri S Robison J Pflugeisen C Kordas G Amram O. Racial and socioeconomic inequities in breast cancer screening before and during the COVID-19 pandemic: analysis of two cohorts of women 50 years +. Breast Cancer. (2022) 29:740–6. doi: 10.1007/s12282-022-01352-2, PMID: 35366175 PMC8976168

[76] Padela AI Murrar S Adviento B Liao C Hosseinian Z Peek M . Associations between religion-related factors and breast cancer screening among American Muslims. J Immigr Minor Health. (2015) 17:660–9. doi: 10.1007/s10903-014-0014-y, PMID: 24700026 PMC4646415

[77] Paranjpe A Zheng C Chagpar AB. Disparities in breast cancer screening between Caucasian and Asian American women. J Surg Res. (2022) 277:110–5. doi: 10.1016/j.jss.2022.03.032, PMID: 35489215

[78] Sabatino SA Thompson TD Guy GP de Moor JS Tangka FK. Mammography use among medicare beneficiaries after elimination of cost sharing. Med Care. (2016) 54:394–9. doi: 10.1097/mlr.0000000000000495, PMID: 26759983 PMC5863723

[79] Schommer L Mikulski MF Goodgame B Brown KM. Racial disparities in breast cancer presentation and diagnosis in COVID-era Central Texas. J Surg Res. (2023) 288:79–86. doi: 10.1016/j.jss.2023.02.021, PMID: 36948036 PMC10026721

[80] Sealy-Jefferson S Roseland ME Cote ML Lehman A Whitsel EA Mustafaa FN . Rural-urban residence and stage at breast cancer diagnosis among postmenopausal women: the Women’s health initiative. J Women's Health. (2019) 28:276–83. doi: 10.1089/jwh.2017.6884, PMID: 30230942 PMC6909717

[81] Shon E-J Townsend AL. Predictors of never having a mammogram among Chinese, Vietnamese, and Korean immigrant women in the U.S. PLoS One. (2019) 14:e0224505. doi: 10.1371/journal.pone.0224505, PMID: 31693678 PMC6834271

[82] Spada NG Geramita EM Zamanian M van Londen GJ Sun Z Sabik LM. Changes in disparities in stage of breast cancer diagnosis in Pennsylvania after the affordable care act. J Women's Health. (2021) 30:324–31. doi: 10.1089/jwh.2020.8478, PMID: 32986501 PMC7957377

[83] Tangka FK Subramanian S Mobley LR Hoover S Wang J Hall IJ . Racial and ethnic disparities among state Medicaid programs for breast cancer screening. Prev Med. (2017) 102:59–64. doi: 10.1016/j.ypmed.2017.06.024, PMID: 28647544 PMC5840870

[84] Thomas M James M Vittinghoff E Creasman JM Schillinger D Mangurian C. Mammography among women with severe mental illness: exploring disparities through a large retrospective cohort study. Psychiatr Serv. (2018) 69:48–54. doi: 10.1176/appi.ps.201600170, PMID: 28945184

[85] Tran L Tran P. US urban-rural disparities in breast cancer-screening practices at the national, regional, and state level, 2012-2016. Cancer Causes Control. (2019) 30:1045–55. doi: 10.1007/s10552-019-01217-8, PMID: 31428890

[86] Vang S Margolies LR Jandorf L. Screening mammogram adherence in medically underserved women: does language preference matter? J Cancer Educ. (2022) 37:1076–82. doi: 10.1007/s13187-020-01922-y, PMID: 33169336 PMC8106692

[87] Wang H Gregg A Qiu F Kim J Chen B Wan N . Breast cancer screening for patients of rural accountable care organization clinics: a multi-level analysis of barriers and facilitators. J Community Health. (2018) 43:248–58. doi: 10.1007/s10900-017-0412-x, PMID: 28861654

[88] Wu AM Morse AR Seiple WH Talwar N Hansen SO Lee PP . Reduced mammography screening for breast cancer among women with visual impairment. Ophthalmology. (2021) 128:317–23. doi: 10.1016/j.ophtha.2020.07.029, PMID: 32682837 PMC9675615

[89] Kadivar H Kenzik KM Dewalt DA Huang I-C. The association of English functional health literacy and the receipt of mammography among Hispanic women compared to non-Hispanic U.S.-born white women. PLoS One. (2016) 11:e0164307. doi: 10.1371/journal.pone.0164307, PMID: 27732660 PMC5061417

[90] Ryu SY Crespi CM Maxwell AE. What factors explain disparities in mammography rates among Asian-American immigrant women? A population-based study in California. Womens Health Issues. (2013) 23:e403–10. doi: 10.1016/j.whi.2013.08.005, PMID: 24183415 PMC3833860

[91] Korn AR Walsh-Bailey C Correa-Mendez M DelNero P Pilar M Sandler B . Social determinants of health and US cancer screening interventions: A systematic review. CA Cancer J Clin. (2023) 73:461–79. doi: 10.3322/caac.21801 37329257 PMC10529377

[92] Coughlin SS. Social determinants of breast cancer risk, stage, and survival. Breast Cancer Res Treat. (2019) 177:537–48. doi: 10.1007/s10549-019-05340-7 31270761

[93] Alcaraz KI Wiedt TL Daniels EC Yabroff KR Guerra CE Wender RC. Understanding and addressing social determinants to advance cancer health equity in the United States: a blueprint for practice, research, and policy. CA Cancer J Clin. (2020) 70:31–46. doi: 10.3322/caac.21586, PMID: 31661164

[94] Gomez SL Shariff-Marco S DeRouen M Keegan TH Yen IH Mujahid M . The impact of neighborhood social and built environment factors across the cancer continuum: current research, methodological considerations, and future directions. Cancer. (2015) 121:2314–30. doi: 10.1002/cncr.29345, PMID: 25847484 PMC4490083

[95] Williams AD Moo TA. The impact of socioeconomic status and social determinants of health on disparities in breast Cancer incidence, treatment, and outcomes. Curr Breast Cancer Rep. (2023) 15:30–6. doi: 10.1007/s12609-023-00473-7

[96] Sabik LM Vichare AM Dahman B Bradley CJ. Co-payment policies and breast and cervical cancer screening in Medicaid. Am J Manag Care. (2020) 26:69–74. doi: 10.37765/ajmc.2020.42395, PMID: 32059094 PMC8011838

[97] Trivedi AN Rakowski W Ayanian JZ. Effect of cost sharing on screening mammography in Medicare health plans. N Engl J Med. (2008) 358:375–83. doi: 10.1056/NEJMsa070929, PMID: 18216358

[98] Amin A Shriver CD Henry LR Lenington S Peoples GE Stojadinovic A. Breast cancer screening compliance among young women in a free access healthcare system. J Surg Oncol. (2008) 97:20–4. doi: 10.1002/jso.20895, PMID: 17918226

[99] Koç H O’Donnell O Van Ourti T. “Conclusion: In the absence of a universal screening program in the U.S., determinants of access—income, insurance coverage and receipt of medical advice—appear to drive the education disparities in screening mammography.” (2018) Available at: https://www.ncbi.nlm.nih.gov/pmc/articles/PMC6163342/

[100] White A Thompson TD White MC Sabatino SA de Moor J Doria-Rose PV . Cancer screening test use—United States, 2015. MMWR Morb Mortal Wkly Rep. (2017) 66:201–6. doi: 10.15585/mmwr.mm6608a1, PMID: 28253225 PMC5657895

[101] Tsapatsaris A Babagbemi K Reichman MB. Barriers to breast cancer screening are worsened amidst COVID-19 pandemic: a review. Clin Imaging. (2022) 82:224–7. doi: 10.1016/j.clinimag.2021.11.025, PMID: 34896935 PMC8648670

[102] Karliner LS Ma L Hofmann M Kerlikowske K. Language barriers, location of care, and delays in follow-up of abnormal mammograms. Med Care. (2012) 50:171–8. doi: 10.1097/MLR.0b013e31822dcf2d, PMID: 21993060 PMC3918470

[103] Sentell TL Tsoh JY Davis T Davis J Braun KL. Low health literacy and cancer screening among Chinese americans in California: a cross-sectional analysis. BMJ Open. (2015) 5:e006104. doi: 10.1136/bmjopen-2014-006104, PMID: 25564140 PMC4289731

[104] Baccolini V Isonne C Salerno C Giffi M Migliara G Mazzalai E . The association between adherence to cancer screening programs and health literacy: a systematic review and meta-analysis. Prev Med. (2022) 155:106927. doi: 10.1016/j.ypmed.2021.106927, PMID: 34954244

[105] Liang W Wang JH Chen M-Y Feng S Lee M Schwartz MD . Developing and validating a measure of Chinese cultural views of health and cancer. Health Educ Behav. (2008) 35:361–75. doi: 10.1177/1090198106294893 17602102

[106] Molaei-Zardanjani M Savabi-Esfahani M Taleghani F. Fatalism in breast cancer and performing mammography on women with or without a family history of breast cancer. BMC Womens Health. (2019) 19:116. doi: 10.1186/s12905-019-0810-6, PMID: 31519195 PMC6743202

[107] Barton MB Moore S Shtatland E Bright R. The relation of household income to mammography utilization in a prepaid health care system. J Gen Intern Med. (2001) 16:200–3. doi: 10.1111/j.1525-1497.2001.00228.x, PMID: 11318916 PMC1495187

[108] Mahmood A Kedia S Dillon PJ Kim H Arshad H Ray M. Food security status and breast cancer screening among women in the United States: evidence from the health and retirement study and health care and nutrition study. Cancer Causes Control. (2023) 34:321–35. doi: 10.1007/s10552-023-01667-1, PMID: 36695824

[109] Damiani G Basso D Acampora A Bianchi CB Silvestrini G Frisicale EM . The impact of level of education on adherence to breast and cervical cancer screening: evidence from a systematic review and meta-analysis. Prev Med. (2015) 81:281–9. doi: 10.1016/j.ypmed.2015.09.011, PMID: 26408405

[110] Khalil S Hatch L Price CR Palakurty SH Simoneit E Radisic A . Addressing breast Cancer screening disparities among uninsured and insured patients: a student-run free clinic initiative. J Community Health. (2020) 45:501–5. doi: 10.1007/s10900-019-00767-x, PMID: 31667647

[111] Lawson MB Lee CI Hippe DS Chennupati S Fedorenko CR Malone KE . Receipt of screening mammography by insured women diagnosed with breast cancer and impact on outcomes. J Natl Compr Cancer Netw. (2021) 19:1156–64. doi: 10.6004/jnccn.2020.7801, PMID: 34330103

